# PARP14 is a PARP with both ADP-ribosyl transferase and hydrolase activities

**DOI:** 10.1126/sciadv.adi2687

**Published:** 2023-09-13

**Authors:** Nina Đukić, Øyvind Strømland, Jonas Damgaard Elsborg, Deeksha Munnur, Kang Zhu, Marion Schuller, Chatrin Chatrin, Pulak Kar, Lena Duma, Osamu Suyari, Johannes Gregor Matthias Rack, Domagoj Baretić, Dorian Richard Kenneth Crudgington, Joséphine Groslambert, Gerissa Fowler, Sven Wijngaarden, Evgeniia Prokhorova, Jan Rehwinkel, Herwig Schüler, Dmitri V. Filippov, Sumana Sanyal, Dragana Ahel, Michael L Nielsen, Rebecca Smith, Ivan Ahel

**Affiliations:** ^1^ Sir William Dunn School of Pathology, University of Oxford, Oxford OX1 3RE, UK.; ^2^ Department of Biomedicine, University of Bergen, 5020 Bergen, Norway.; ^3^ Proteomics Program, Novo Nordisk Foundation Center for Protein Research, Faculty of Health and Medical Sciences, University of Copenhagen, Blegdamsvej 3B, 2200 Copenhagen, Denmark.; ^4^ MRC Centre for Medical Mycology, University of Exeter, Geoffrey Pope Building, Stocker Road, Exeter EX4 4QD, UK.; ^5^ Medical Research Council Human Immunology Unit, Medical Research Council Weatherall Institute of Molecular Medicine, Radcliffe Department of Medicine, University of Oxford, Oxford OX3 9DS, UK.; ^6^ Leiden Institute of Chemistry, Leiden University, Einsteinweg 55, 2333 CC Leiden, Netherlands.; ^7^ Center for Molecular Protein Science, Department of Chemistry, Lund University, 22100 Lund, Sweden.

## Abstract

PARP14 is a mono–ADP-ribosyl transferase involved in the control of immunity, transcription, and DNA replication stress management. However, little is known about the ADP-ribosylation activity of PARP14, including its substrate specificity or how PARP14-dependent ADP-ribosylation is reversed. We show that PARP14 is a dual-function enzyme with both ADP-ribosyl transferase and hydrolase activity acting on both protein and nucleic acid substrates. In particular, we show that the PARP14 macrodomain 1 is an active ADP-ribosyl hydrolase. We also demonstrate hydrolytic activity for the first macrodomain of PARP9. We reveal that expression of a PARP14 mutant with the inactivated macrodomain 1 results in a marked increase in mono(ADP-ribosyl)ation of proteins in human cells, including PARP14 itself and antiviral PARP13, and displays specific cellular phenotypes. Moreover, we demonstrate that the closely related hydrolytically active macrodomain of SARS2 Nsp3, Mac1, efficiently reverses PARP14 ADP-ribosylation in vitro and in cells, supporting the evolution of viral macrodomains to counteract PARP14-mediated antiviral response.

## Teaser

PARP14 is an antiviral PARP that combines ADP-ribosylation writer, reader and eraser functions in one polypeptide.

## INTRODUCTION

Cells must quickly adapt to both internal and external changes. This could be due to internal pressures such as changes in metabolic demands that require alteration in transcription or protein translation, DNA replication, or DNA damage repair, as well as from external pressures such as invasion of pathogenic bacteria and viruses. For such changes to occur efficiently, cells have developed a number of signaling pathways to transduce signals rapidly. This often involves posttranscriptional or posttranslational modification (PTM) of nucleic acids and proteins, respectively. One such signaling type is the adenosine 5′-diphosphate (ADP)–ribosylation (ADPr). ADPr has been shown to target both proteins (*1*) and nucleic acids, including RNA and DNA (*2*), with modification on proteins able to occur on different amino acid acceptors including serine, glutamate, and arginine (*3*–*5*).

Like in most signaling pathways, there are proteins required for the writing, reading, and reversal of ADPr. The largest known family of proteins that are responsible for the addition of ADP-ribose to proteins or nucleic acids are the poly(ADP-ribose) polymerases (PARPs). PARPs function by transferring ADP-ribose from NAD^+^ [nicotinamide adenine dinucleotide (oxidized form)] onto a target, releasing nicotinamide. These ADP-ribose moieties can either be added as a single moiety, known as mono-ADPr, or they can be attached as long branched polymers, so called poly(ADP-ribosyl)ation (*6*). A number of different reader domains have been shown to recognize and bind ADP-ribose including macrodomains that primarily recognize either mono–ADP-ribose or the terminal ADP-ribose moiety of a polymer or WWE domains and PAR-binding zinc fingers that specifically recognize poly(ADP-ribose) (*7*–*10*). Last, there are eraser proteins involved in the removal of ADP-ribose, primarily ADP-ribosyl hydrolases (ARHs), or hydrolytic macrodomains, of which four—poly(ADP-ribosyl) glycohydrolase (PARG), MacroD1, MacroD2, and terminal ADP-ribose protein glycohydrolase (TARG1)—have been described in humans (*11*).

While there have been strides taken in understanding how several members of the PARP family function, there are still outstanding questions regarding the specificity of their ADPr activity, as well as the hydrolases that remove their modification. The best understood aspect of ADPr signaling in humans is its role in the DNA damage response. Here, PARP1 or PARP2 will recognize and bind to DNA breaks (*12*) where they interact with their auxiliary factor histone PARylation factor 1 (HPF1) (*13*). Together, they ADP-ribosylate proteins around the break site, including PARP1 itself, on serine residues (*14*, *15*). The modified proteins can be also then recognized by ADP-ribose–binding repair proteins such as ALC1, XRCC1, and APLF, which are required for efficient DNA repair (*16*). Following the initial ADP-ribose signaling at DNA breaks, the ADP-ribose moieties are efficiently removed by PARG, which degrades long polymers of ADP-ribose, while the final ADP-ribose on serine residues is removed by ARH3 (*17*). Recently, we have also begun to understand the complexity of ADPr signaling on nucleic acids in bacterial systems, where a PARP-like protein, DarT2, catalyses the ADPr of thymidine bases, which can be efficiently reversed by the hydrolytic macrodomain DarG (*18*, *19*). Several human PARPs have been suggested to ADP-ribosylate 5′ and 3′ phosphorylated single-stranded (ss) DNA and RNA (*2*, *20*–*22*). This is reversed by endogenous ADP-ribosyl hydrolases, including PARG, TARG1, MacroD1, MacroD2, and ARH3 (*20*, *21*).

Despite the wealth of knowledge on ADPr and PARP1, much less is known about most of the other human PARPs. One poorly understood subgroup is the interferon-induced “antiviral PARPs,” which include PARP7, 9, 10, 11, 12, 13, 14, and 15. Several members of this class have been reported to modify both protein and nucleic acid substrates (*20*–*22*), while PARP9 and PARP13 are reported to be catalytically inactive (*3*). These antiviral PARPs are interferon-inducible and were shown to confer resistance to a range of viruses, including coronaviruses, influenza, HIV, and Ebola (*23*–*27*). These PARPs are also under positive evolutionary selection, strongly suggesting coevolution with viruses as a consequence of host-virus conflicts (*28*).

**Fig. 1.**
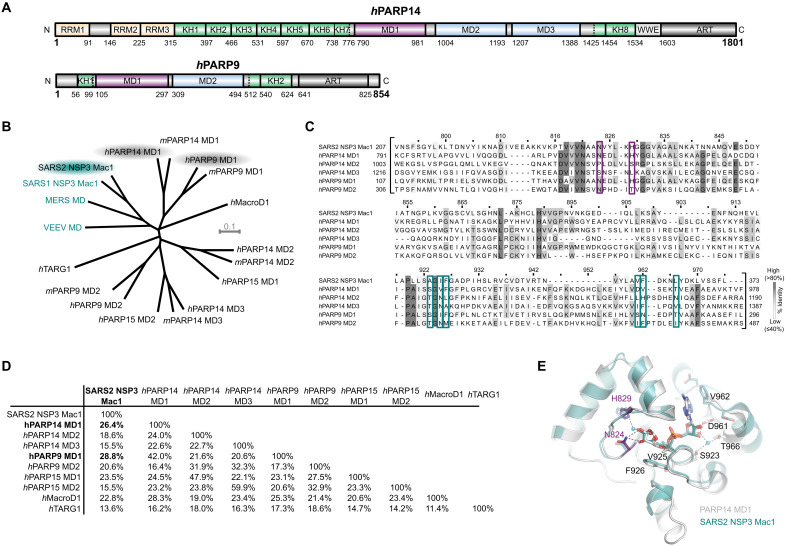
PARP14 and PARP9 macrodomain 1 are similar to SARS2 Mac1. (**A**) Domain architecture of human PARP14 and PARP9. (**B**) Unrooted phylogenetic tree of human and mouse macrodomains including of PARP9, 14, and 15 and viral macrodomains (highlighted in cyan). (**C**) Multiple sequence alignment showing conservation of catalytic residues (magenta-framed) and residues involved in ADP-ribose coordination (cyan-framed) of human PARP14 and PARP9 macrodomains in comparison to SARS2 Nsp3 Mac1. Numbers on top of the residues refer to human PARP14 MD1. (**D**) Pairwise sequence identity comparison of SARS2 Nsp3 Mac1 and human macrodomains. (**E**) Crystal structure overlay of PARP14 MD1 [Protein Data Bank (PDB) ID: 3Q6Z] and SARS2 Nsp3 Mac1 (PDB ID: 7KQP) both in complex with ADP-ribose (root mean square deviation of 1.02 Å over 214 C^α^). The catalytic residues are highlighted in magenta.

The largest of all the human PARPs is PARP14. The N terminus of PARP14 harbors three RRM (RNA recognition motif) domains and eight putative K homology (KH) domains, which may bind RNA or DNA, in addition to three ADPr-binding macrodomains (MD1, MD2, MD3) (Fig. 1A) (*29*). The C terminus of PARP14 contains a WWE domain, followed by the catalytic ADP-ribosyl transferase (ART) domain. PARP14 has been reported to regulate several different pathways involved in immunity, inflammation, and genome stability. PARP14 was initially shown to be involved in transcriptional regulation, acting as a molecular switch of interleukin-4 (IL-4)–regulated genes (*30*). In basal conditions, PARP14 represses gene transcription by binding to IL-4–responsive promoters and recruiting histone deacetylase 2/3 (HDAC2/3). In contrast, under IL-4–stimulated conditions, PARP14 is activated, leading to the dissociation of HDAC2/3 from the promoter regions. Consequently, this allows the binding of the transcription factor signal transducer and activator of transcription 6 (STAT6), as well as other transcription cofactors, to their target genes and allows efficient gene transcription (*31*). PARP14 has also been shown to regulate transcription in response to interferon-γ (IFN-γ) stimulation. Specifically, PARP14 has been suggested to ADP-ribosylate STAT1, inhibiting phosphorylation of STAT1 and subsequent activation of proinflammatory gene expression. With its role in regulating genomic stability, PARP14 was shown to regulate DNA repair by homologous recombination (HR) and in the replication stress response (*32*–*34*).

PARP14 has also been reported to play an important role in the antiviral response (*25*, *35*). In the context of coronavirus infection, PARP14 is required to enhance type I interferon production and restrict replication of murine hepatitis virus, a model coronavirus (*25*). To combat the antiviral activity of these PARPs, severe acute respiratory syndrome coronavirus (SARS-CoV) contains a hydrolytic macrodomain (*36*, *37*) within the nonstructural protein 3 (Nsp3), which has been suggested to remove PARP14-mediated ADPr (*38*). Nsp3 macrodomain 1 (Mac1) is critical for virus replication in vivo and viruses with mutated or absent macrodomains are unable to hydrolyse host ADPr and therefore associated with reduced viral loads and increased sensitivity to IFN-I treatment in PARP14-proficient cells (*25*, *26*). However, the exact mechanism of activation and the molecular substrates of PARP14 and the viral macrodomains are still poorly characterized. Notably, PARP14 has been shown to efficiently modify itself both in its catalytic region and isolated MD2 and MD3, but not MD1 (*3*, *38*), and ADPr of endogenous PARP14 on acidic residues has also been detected in IFN-γ–stimulated primary human macrophages (*39*).

PARP14’s close relative, PARP9, is also induced by interferon stimulation and is expressed from the same genetic locus as PARP14 (chromosome 3q21.1). Structurally, PARP9 is similar to PARP14 with two KH domains and two macrodomains at the N terminus and a C-terminal ART domain (Fig. 1A). No ART activity of PARP9 has been detected to date, presumably because of the lack of several essential catalytic amino acids within the ART domain (*3*). However, PARP9 forms a heterodimer with Deltex E3 ubiquitin ligase 3L (DTX3L), which ubiquitinates different cellular, as well as viral substrates. For instance, ubiquitination of histones promotes increased histone methylation, leading to chromatin remodeling and eventually enhanced expression of interferon-stimulated genes (*40*). PARP9 has also been reported to serve as an RNA virus sensor, leading to activation of the phosphatidylinositol 3-kinase/AKT3 signaling pathway and subsequent production type I IFN (*41*). In addition to roles in viral defense, PARP9/DTX3L has also been implicated in the maintenance of genome integrity. Specifically, PARP9/DTX3L is recruited to sites of damage where DTX3L ubiquitinates different targets, including p53 (*42*, *43*). Recently, it was reported that DTX3L can also ubiquitinate ADP-ribose on proteins in vitro, presenting a PTM with unknown physiological functions (*44*).

Previous studies have suggested that Mac1 of SARS-CoV-2 (SARS2) Nsp3 is closely related to macrodomain 1 of PARP9 and PARP14 (*38*). Given these similarities, we sought to examine whether PARP9 and PARP14 MD1 share the same hydrolytic function. Here, we show that these macrodomains are active hydrolases and can remove ADPr from both protein and nucleic acid substrates. We also show that PARP14 modifies proteins in cells by mono-ADPr and that this ADPr is reversed by its own macrodomain. In addition, we identify targets of PARP14 MD1 by mass spectrometry (MS). Thus, we show that PARP14 is a PARP that acts both as a transferase and as a hydrolase. We further show that SARS2 Mac1 can reverse PARP14-dependent ADPr.

## RESULTS

### PARP14 and PARP9 macrodomain 1 exhibit ADP-ribosyl hydrolase activity on protein substrates

PARP14, the largest of the human PARPs belonging to the macrodomain-containing PARPs, together with PARP9 and PARP15, is a potent mono (ADP-ribosyl) transferase (Fig. 1A) (*3*, *29*, *38*, *45*). PARP14 ADPr activity is efficiently reversed in vitro by SARS2 Nsp3 Mac1 (*38*); however, human endogenous hydrolases that can reverse PARP14 modification remain elusive. To identify potential human hydrolases with activity toward PARP14-mediated ADPr, we compared human macrodomains to Mac1. Phylogenetic analysis suggests that while there is an obvious homology to the known hydrolases such as MacroD1, the closest orthologs are the first macrodomain of PARP14 (PARP14 MD1) and the first macrodomain of PARP9 (PARP9 MD1) (Fig. 1, B to D), while macrodomains 2 (MD2) and 3 (MD3) of PARP14 and macrodomain 2 (MD2) of PARP9 are more diverged. Comparison of ADP-ribose complex structures of PARP14 MD1 and Mac1 reveals that the residues important for distal ribose coordination are also structurally conserved, suggesting that PARP14 MD1 and Mac1 potentially share the same catalytic ability and functional context (Fig. 1, D and E).

**Fig. 2.**
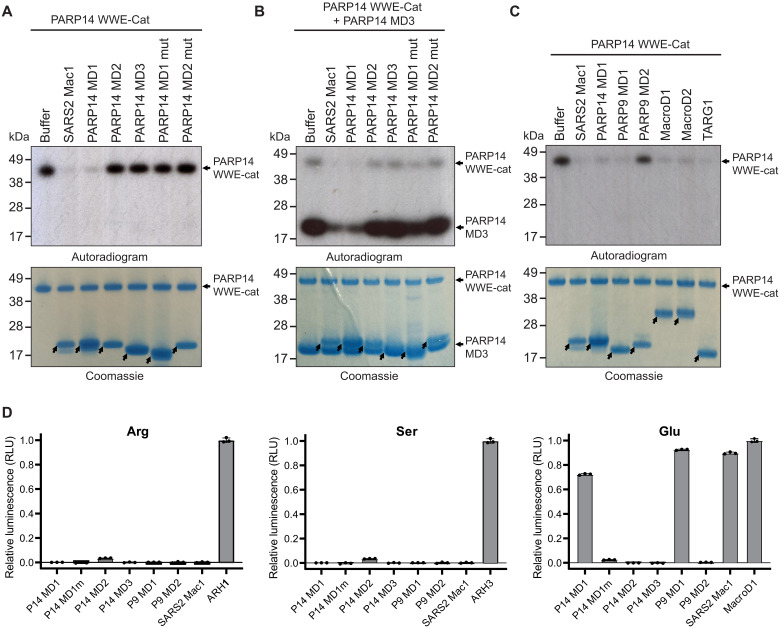
PARP14 and PARP9 MD1 reverse glutamate-linked PARP14 auto- and trans-ADPr. (**A**) PARP14 WWE-CAT was auto–ADP-ribosylated using NAD^+^ spiked with ^32^P NAD^+^. The ADP-ribosyl hydrolysis activity of PARP14 MD1, MD2, MD3, MD1mut (G823E), MD2mut (G1044E), and SARS2 Nsp3 Mac1 (SARS2 Mac1) was assessed upon incubation with automodified PARP14 WWE-CAT. (**B**) PARP14 WWE-CAT and PARP14 MD3 were auto– and trans–ADP-ribosylated, respectively, using NAD^+^ spiked with ^32^P NAD^+^. The trans-ADPr hydrolysis activity of PARP14 MD1, MD2, MD3, MD1mut, MD2mut, and SARS2 Mac1 was assessed upon incubation with the trans-modified PARP14 MD3 and auto-modified PARP14 WWE-CAT. (**C**) PARP14 WWE-CAT was auto–ADP-ribosylated using NAD^+^ spiked with ^32^P NAD^+^. The ADP-ribosyl hydrolysis activity of SARS2 Mac1, PARP14 MD1, PARP9 MD1, PARP9 MD2, MacroD1, MacroD2, and TARG1 was determined upon incubation with automodified PARP14 WWE-CAT. Samples in (A to C) were analyzed by Coomassie brilliant blue staining and autoradiography. The arrows show the position of the indicated macrodomains. (**D**) Hydrolysis of arginine-, serine-, and glutamate-linked mono(ADP-ribosyl)ation on synthetic peptides by PARP14 MD1, PARP14 MD1mut, PARP14 MD2, PARP14 MD3, PARP9 MD1, PARP9 MD2, and SARS2 Mac1. Briefly, the released ADP-ribose was converted by NUDT5 to adenosine 5′-monophosphate (AMP), which subsequently was detected by luminescence using the AMP-Glo assay (Promega). Samples are background-corrected and normalized to the positive control, ARH1 for arginine, ARH3 for serine, and MacroD1 for glutamate. The data represent means ± SD measured in triplicates.

These observations prompted us to investigate the potential ADP-ribosyl hydrolase activities of the macrodomains in PARP9 and PARP14. First, we used the automodified PARP14 catalytic fragment (WWE-CAT) as a model substrate and the isolated macrodomains derived from PARP14 (MD1 to MD3) as described previously (*38*). PARP14 MD1 and SARS2 Mac1 have a notable hydrolysis activity on automodified PARP14 WWE-CAT, while MD2 and MD3 did not exhibit activity (Fig. 2A and fig. S1A). To strengthen our finding, we introduced a point mutation G832E in PARP14 MD1 that sterically blocks the active site in macrodomain hydrolases for ADP-ribose binding (*46*). As predicted the mutation diminished the catalytic activity of PARP14 MD1 (Fig. 2A). Mutating the corresponding residue in PARP14 MD2 had no discernible effect (Fig. 2A). PARP14 MD3 has also been reported to be robustly modified by PARP14 (*38*). We used this to our advantage and examined whether PARP14 MD1 could hydrolyse trans-modified PARP14 MD3. We were able to observe that PARP14 MD1 and SARS2 Nsp3 Mac1 efficiently reversed PARP14 MD3 ADPr (Fig. 2B), while PARP14 MD2 and PARP14 MD3 had no discernible effect on the ADP-ribosylation level of trans-modified PARP14 MD3.

PARP14 is involved in macrophage activation whereby gene expression enabling the defense against pathogens is induced. In particular, PARP14 has been suggested to ADP-ribosylate STAT1α, preventing STAT1α phosphorylation, which is essential for STAT1α to drive transcription of proinflammatory genes (*47*). Conversely, PARP9 has been reported to antagonize the activation by inhibiting the ADPr of STAT1α (*47*). Given the similarity between PARP9 MD1, PARP14 MD1, and SARS2 Mac1 and the reported role of PARP9 in antagonizing PARP14 ADPr, we investigated whether PARP9 MD1 can reverse PARP14 automodification. PARP9 MD1 could efficiently reverse PARP14 ADPr, while PARP9 MD2 had no observable effect (Fig. 2C). We also tested several known human hydrolases and saw that MacroD1, MacroD2, and TARG1, known to remove glutamate-linked ADP-ribose from target proteins (*48*, *49*), exhibited pronounced hydrolytic activity on automodified PARP14 (Fig. 2C), suggesting that the PARP14-derived ADPr could be glutamate linked. To investigate the amino acid specificity of the PARP9 MD1 and PARP14 MD1, we assessed their activity against chemically synthesized defined ADP-ribosylated peptide substrates modified on serine, arginine, and glutamate, respectively (Fig. 2D).We observed that PARP14 MD1 and PARP9 MD1 are specifically active on glutamate-ADPr, similar to SARS2 Mac1 and MacroD1. PARP14 MD1 and PARP9 MD1 showed no activity on serine- and arginine-linked peptides in contrast to the cognate hydrolases ARH3 (*17*) and ARH1 (*50*).

### PARP14 and PARP9 macrodomain 1 exhibit ADP-ribosylhydrolase activity on nucleic acid substrates

**Fig. 3.**
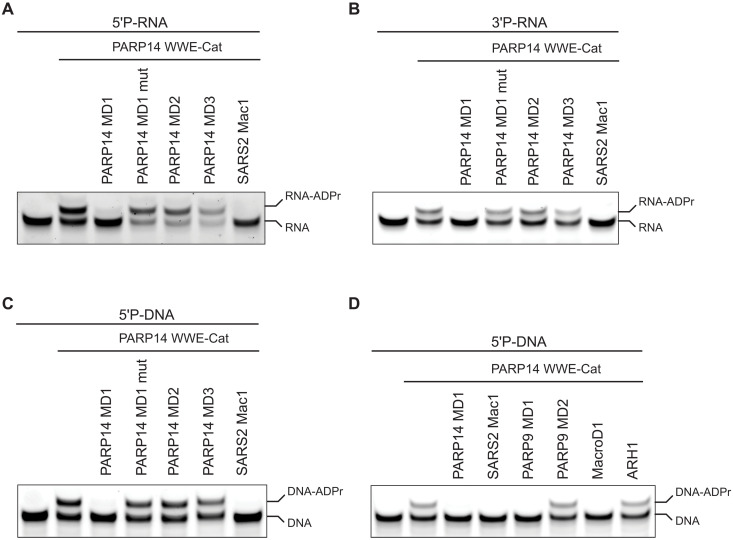
PARP14 and PARP9 MD1 reverse ADPr of ssRNA and ssDNA. (**A**) ssRNA with 5′ phosphate and 3′ cyanine 3 (Cy3), (**B**) ssRNA with 3′ phosphate and 5′ Cy3, and (**C**) ssDNA with 5′ phosphate and 3′ Cy3 were ADP-ribosylated using PARP14 WWE-CAT. Subsequently, the ADPr was hydrolyzed by treating the modified oligos with PARP14 MD1, MD1mut, MD2, MD3, or SARS2 Mac1. (**D**) ssDNA with 5′ phosphate and 3′ Cy3 was ADP-ribosylated using PARP14 WWE-CAT. Following, the ADP ribose modification was hydrolyzed by subjecting the ADP-ribosylated oligo to PARP14 MD1, SARS2 Mac1, PARP9 MD1 and MD2, MacroD1, or ARH1.

The domain architecture of PARP14 suggests that it is tightly linked to nucleic acids because it harbors three RRM domains and eight KH domains that all are putative ssRNA or ssDNA binders (Fig. 1A). Furthermore, PARP14 is interferon induced and plays a role in the innate immune response against viruses (*51*). Interferon-induced antiviral PARPs, such as PARP10 and PARP11, ADP-ribosylate 5′ and 3′ phosphorylated ssRNA and ssDNA (*21*). On the basis of these observations, we postulated that PARP14 MD1 could reverse ssDNA and ssRNA ADPr. ssRNAs phosphorylated at either the 5′ or at 3′ were ADP-ribosylated with PARP14, reaction-inhibited with PARP14i (RBN012759), and then used as potential substrates for PARP14 macrodomains [Fig. 3, A to C (lane 2)]. PARP14 MD1 efficiently reversed the ADPr from both RNA substrates, and as expected, PARP14 MD2 and MD3 did not (Fig. 3, A and B). SARS2 Mac1, as reported previously, could also remove the modification (Fig. 1, A and B) (*21*). Next, we tested whether PARP14 MD1 could also reverse the ADPr of 5′ phosphorylated ssDNA. As for ssRNA, PARP14 MD1 reversed the ADPr on ssDNA, while PARP14 MD2 and MD3 did not (Fig. 3C and fig S1B). When comparing hydrolase efficiencies against ADP-ribosylated DNA or RNA, we did not observe major differences in PARP14 activity for either substrate (fig S1C). We also determined whether PARP9 MD1 can reverse ADPr of 5′ phosphorylated ssDNA, finding that PARP9 MD1 but not MD2 reversed the modification (Fig. 3D). As reported previously, MacroD1 could also remove ADPr of 5′ phosphorylated ssDNA, while the unrelated human hydrolase ARH1 could not. Last, we tested whether PARP14 was capable of adding and removing ADPr on 5′ or 3′ phosphorylated double-stranded DNA (dsDNA). Our data show that PARP14 modifies dsDNA substrates including blunt-ended, gapped, nicked, forked, and single-stranded overhangs equally efficiently at 5′ or 3′ phosphates, while PARP14 MD1 more efficiently removes ADPr from 5′ phosphorylated dsDNA substrates (fig. S2). Last, in a competition assay where DNA-ADPr was in eightfold molar excess compared to an ADP-ribosylated protein substrate, we did not see any notable difference in protein-ADPr hydrolysis, suggesting that in our experimental setup, DNA is not an efficient competitor for protein-ADP-ribose hydrolysis (fig. S1A). Together, our data identify two additional ADP-ribosyl hydrolases in humans (MD1 of PARP9 and PARP14) and demonstrate that PARP14 represents a PARP enzyme that can reverse its own modification on both protein and nucleic acid substrates.

### PARP14 shows ADP-ribosyl transferase and hydrolase activity on different cellular substrates

**Fig. 4.**
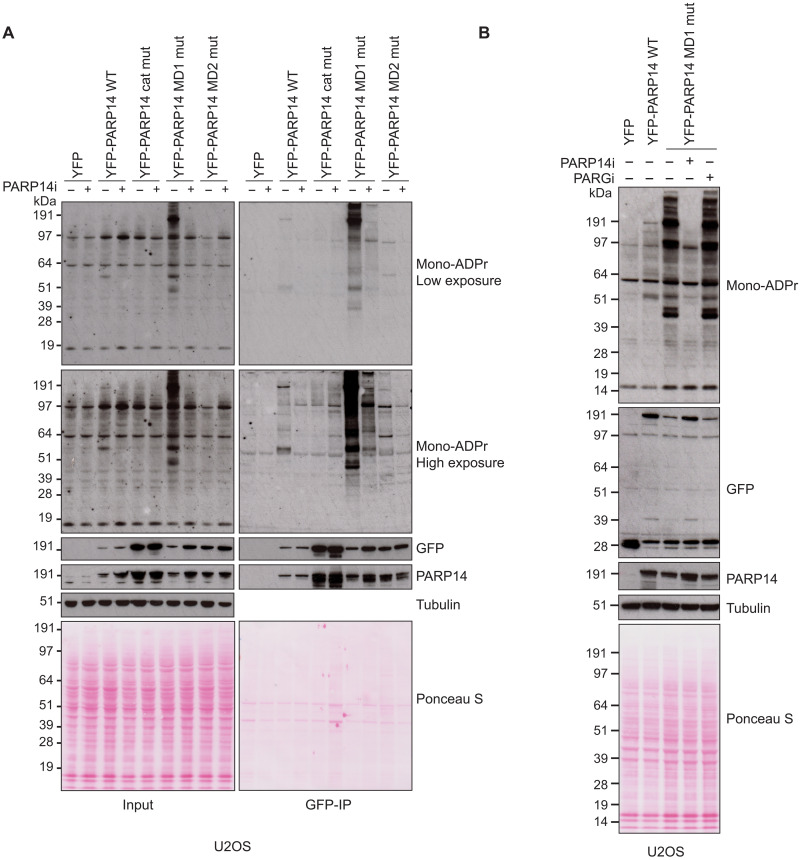
PARP14 ADPr is reversed by its own macrodomain 1. (**A**) U2OS cells were transfected with the indicated plasmids in the presence or absence of PARP14 inhibitor (PARP14i). Cell lysates and green fluorescent protein (GFP)–immunoprecipitations (GFP-IPs) were examined by Western blotting using the indicated antibodies. (**B**) U2OS cells were transfected with the indicated plasmids in the presence of PARP14 or PARG inhibitor. Cell lysates were examined by Western blotting with the indicated antibodies. For all blots, tubulin was used as a loading control.

We next studied PARP14 activity in human cells. To do so, we transiently transfected U2OS cells, which express PARP14 endogenously, or 293T cells, which are naturally deficient in PARP14 (fig S3, A and B) (*52*), with yellow fluorescent protein (YFP)–tagged full-length PARP14 wild type (WT), PARP14 R1699A catalytic mutant, PARP14 G832E MD1, and PARP14 G1044E MD2 mutant, and examined changes in protein mono(ADP-ribosyl)ation in cell extracts using a mono-ADPr–specific antibody (Fig. 4A and fig. S3C) (*53*). In both cell lines, overexpression of WT PARP14, but not the R1699A ADPr-deficient mutant, resulted in a modest increase of mono-ADPr, indicating that this mutant is devoid of catalytic activity. On the other hand, we observed a marked increase in mono(ADP-ribosyl)ation of a variety of protein sizes when we overexpressed the PARP14 MD1 mutant, suggesting that PARP14 modifies a number of different proteins in the cells. In all cases, treatment with a specific PARP14 inhibitor suppressed the increase in mono-ADPr, indicating that ADPr signal is a result of PARP14 catalytic activity. In contrast to protein ADPr, we were unable to detect any nucleic acid ADPr under the same conditions (fig. S3D). The ADPr pattern seen upon overexpression of the PARP14 MD1 mutant suggests that PARP14 modifies several different proteins in the cells, while the hydrolytic activity of MD1 removes these modifications. A pull-down experiment further suggested that the signal around 200 kDa belongs to automodified PARP14. Addition of PARG inhibitor did not affect the PARP14 signal, suggesting that there is no major extension of PARP14-derived mono-ADPr into polymers (Fig. 4B). PARP14 is an IFN-induced protein (fig. S3B) (*35*). To test whether we can detect PARP14-dependent ADPr endogenously, we stimulated an immunogenic cell line, A549, with IFN-γ and compared the pattern of ADP-ribosylated proteins. After stimulation, we could again see several strongly ADP-ribosylated proteins and that this was abolished upon treatment of cells with the PARP14 inhibitor (fig. S3E). Our data show that PARP14 is one of the most robustly active ART of proteins in human cell extracts upon IFN stimulation. Together, these results suggest that PARP14 is a highly active mono-ART for proteins, but the level of ADPr is tightly regulated by its own hydrolytic macrodomain and by induction of immune responses.

### PARP14-derived ADPr can be reversed by hydrolytic macrodomains

**Fig. 5.**
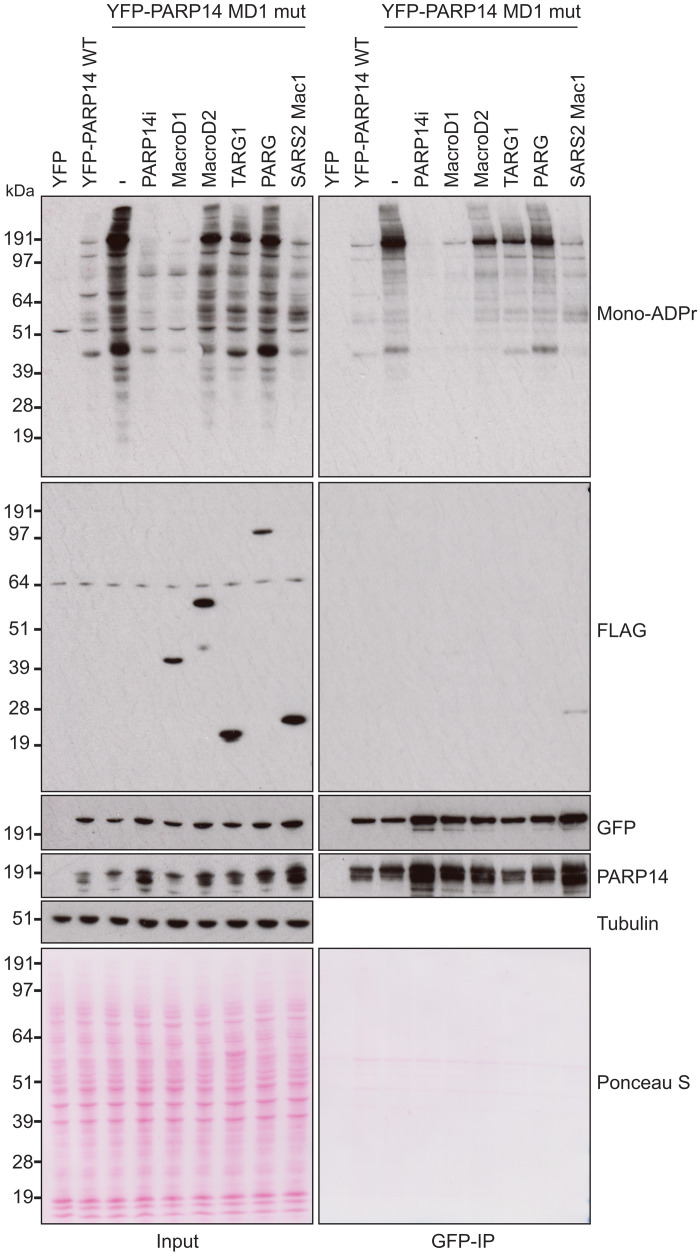
MacroD1 and SARS2 Mac1 can reverse PARP14 ADPr. U2OS cells were transfected with the indicated PARP14 plasmid together in the presence or absence of PARP14i or with FLAG-tagged MacroD1, MacroD2, TARG1, PARG, or SARS2 Mac1. Cell lysates and GFP-IPs were examined by Western blotting using the indicated antibodies. Tubulin was used as a loading control.

While PARP14 MD1 appears to be the dominant hydrolase controlling PARP14-catalyzed ADPr in cells, we also tested whether PARP14 ADPr could be reversed by several other human hydrolases in a cellular context. For this, we coexpressed the PARP14 MD1 mutant, which shows the strongest increase in mono-ADPr signal upon overexpression, together with different FLAG-tagged human hydrolases (Fig. 5). First, we confirmed a strong increase in mono-ADPr upon expression of PARP14 MD1 mutant and that this could be inhibited by treatment with PARP14i. Next, we compared PARP14-dependent ADPr in the presence of the human hydrolases MacroD1, MacroD2, TARG1, and PARG. While MacroD1 could quite efficiently remove PARP14 auto- and trans-ADPr, the activity of MacroD2 and TARG1 could only modestly remove PARP14-dependent ADPr. Last, PARG overexpression did not reduce ADP-ribose, suggesting that PARP14 catalyses largely mono-ADPr as previously suggested (*3*, *38*). These results are consistent with our in vitro data (Fig. 2D) showing that PARP14 auto- and trans-modification targets primarily acidic residues for mono-ADPr and that its modifications are reversed by ADP-ribosyl hydrolases with activity toward acidic residues such as PARP14 MD1 and MacroD1 (*54*).

Given that SARS2 Mac1 hydrolyses PARP14 ADPr in vitro (Fig. 2) (*38*), we wanted to examine whether this also held true in a cellular context. When we coexpressed SARS2 Mac1 together with PARP14 MD1 mutant, we saw a marked reduction in PARP14-derived ADPr (Fig. 5, lane 9), again confirming our results that these two hydrolases, PARP14 MD1 and SARS2 Mac1, can both remove ADPr catalyzed by PARP14. This supports the available genetic data showing that PARP14 and SARS2 Mac1 act as a pair where PARP14-driven ADPr acts to suppress virus proliferation while SARS2 Mac1 counteracts PARP14 antiviral activity through ADP-ribosyl hydrolysis (*25*).

### PARP13 is a target of both ADP-ribosyl transferase and hydrolase activity of PARP14

**Fig. 6.**
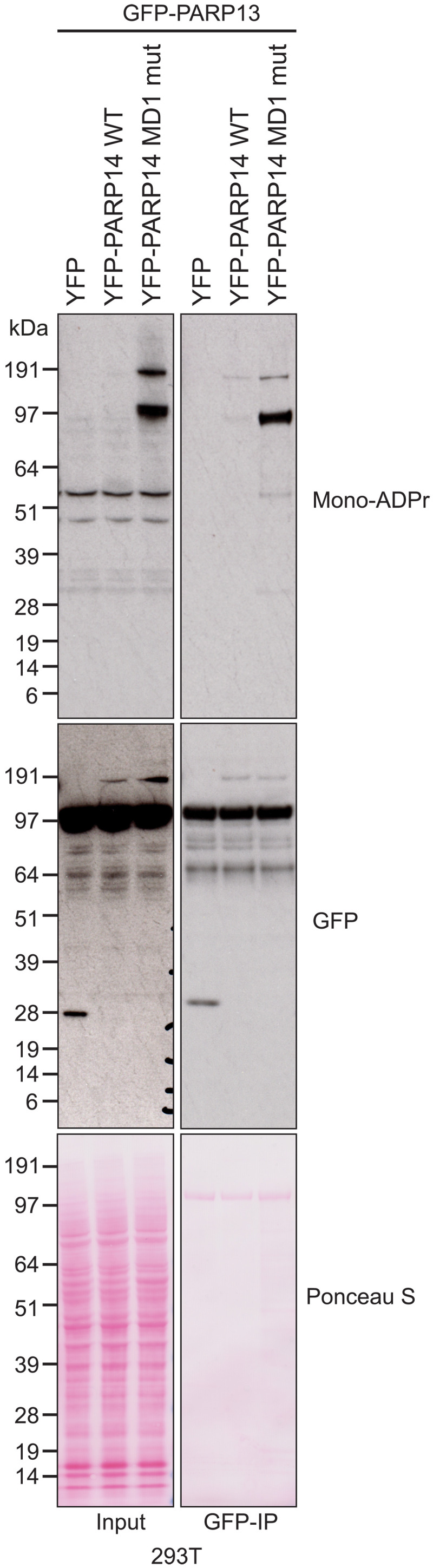
PARP14 acts to add and remove ADPr on PARP13. 293T cells were cotransfected with GFP-PARP13 and the indicated YFP plasmid. Cell lysates and GFP-IPs were examined by Western blotting using the indicated antibodies. Ponceau S staining was used to indicate equal loading.

We then sought to examine the hydrolytic activity of PARP14 MD1 on a known target of PARP14 ADPr. It has previously been reported that PARP14 can modify another antiviral PARP, i.e., PARP13 (*55*). To test this, we coexpressed green fluorescent protein (GFP)–tagged PARP13 with PARP14 WT or PARP14 MD1 mutant (Fig. 6). We observed a strong induction of ADPr on a protein the size of GFP-PARP13 in lysates of cells expressing PARP14 MD1 mutant. We then performed immunoprecipitation to pull-down GFP-PARP13 and examined its ADP-ribosylation state. We found that expression of GFP-PARP13 together with WT PARP14 could modestly increase PARP13 mono-ADPr levels compared to expression with YFP alone. However, PARP13 ADPr was greatly increased upon expression of the PARP14 MD1 mutant. Together, this confirms that PARP14 ADP-ribosylates PARP13 in cells and that the hydrolytic macrodomain MD1 of PARP14 reverses this ADPr.

### MS-based affinity purification reveals ADPr targets regulated by PARP14

**Fig. 7.**
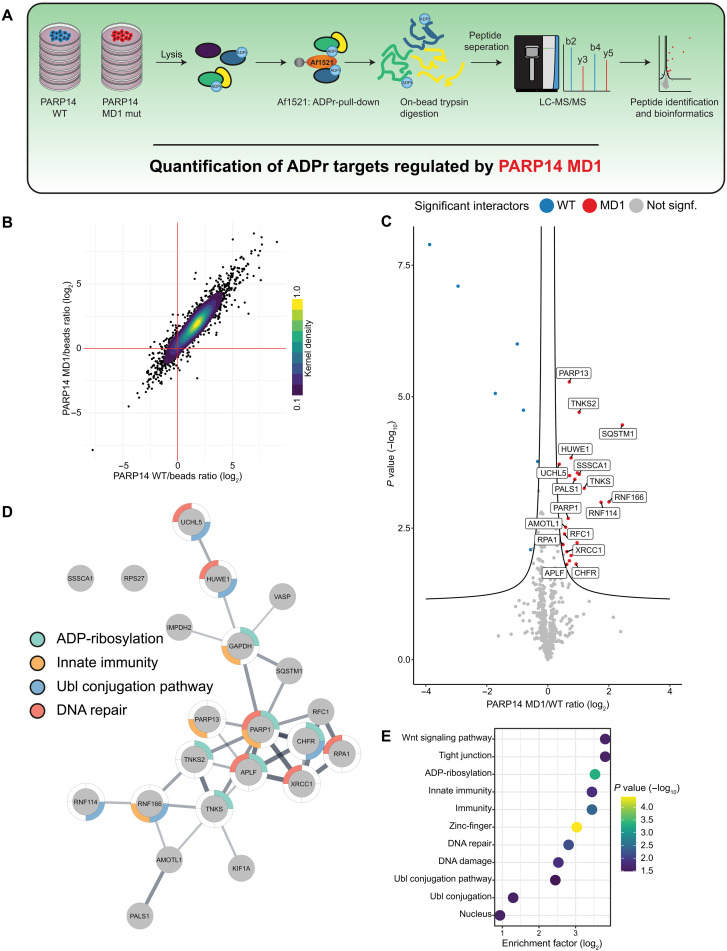
MS identification of ADP-ribosylated proteins regulated by PARP14 macrodomain 1. (**A**) Overview of the experimental design. (**B**) Scatter plot analysis of proteins enriched specifically by the ADPr-binding Af1521 macrodomain. The mean difference in abundance between proteins enriched by the Af1521 compared to control beads for cells expressing WT PARP14 is plotted against cells expressing PARP14 MD1 mutant. The color scale represents the normalized kernel density estimation of the data. (**C**) Analysis of proteins specifically enriched for ADPr in cells expressing PARP14 MD1 mutant compared to WT. The volcano plot shows the sample conditions (*x* axis), plotted against the corresponding *P* value resulting from two-tailed Student’s *t* testing (*y* axis). Proteins significantly down- or up-regulated [false discovery rate (FDR) < 0.05, s0 = 0.1] are represented as blue or red dots, respectively. *N* = 5. (**D**) STRING network visualizing functional interactions (edges) between proteins (nodes) significantly enriched in cells expressing PARP14 MD1 mutant over WT. The thickness of the edges corresponds to their score, and the default STRING clustering confidence score cutoff of 0.4 was used to determine whether two nodes were functionally related. Proteins were colored according to the UniProt keyword displayed on the figure legend if it was significantly enriched. (**E**) Gene set enrichment analysis showing UniProt keywords functionally enriched in the MD1-specific network [highlighted in (D)]. Significantly enriched terms were determined by Fisher exact testing, testing whether categorical terms found in the MD1-specific network were functionally enriched over terms found in the background, which was defined as all proteins significantly enriched by Af1521 over bead controls. Terms were determined to be significant with a Benjamini-Hochberg multiple-hypotheses corrected *P* value <0.05. The terms are ranked by their functional enrichment over the background in descending order and colored by their corresponding Benjamini-Hochberg–adjusted *P* values.

To determine the ADPr targets of the PARP14 and, in particular, of the hydrolytic MD1, we expressed PARP14 WT or PARP14 MD1 mutant in 293T cells and enriched ADP-ribosylated proteins with the Af1521 macrodomain (*56*), a specific ADP-ribose binding domain (Fig. 7A). The enriched proteins were then digested into peptides and analyzed by MS to identify differential binders. Overall, the coefficient of variation was low (<15%) within groups (fig. S4A), and they show a high correlation (fig. S4B), suggesting excellent reproducibility, while distinct clusters can be observed between groups by principal components analysis (fig. S4C).

ADP-ribosylated target proteins were efficiently purified (Fig. 7B), with hundreds of proteins significantly enriched over the background control. As a whole, the majority of the proteins are similarly enriched by the Af1521 macrodomain in PARP14 WT– and PARP14 MD1 mutant–expressing cells (Fig. 7B and fig. S4B). We were able to identify a subset of ADP-ribosylated proteins that were enriched in PARP14 MD1 expressing cells compared to WT, suggesting that their ADPr pattern is regulated by PARP14 MD1 (Fig. 7C). We confirmed the ADPr of the only characterized PARP14 target, PARP13 (Fig. 6) (*55*).

Generally, the ADPr target proteins enriched in cells expressing PARP14 MD1 mutant were functionally highly interconnected (Fig. 7D) and were enriched for terms related to not only ADPr but also ubiquitin signaling, linking processes such as immunity (PARP13, RNF114, and RNF166), DNA repair (RPA1, XRCC1, and PARP1), and tankyrase (TNKS) biology [TNKS1, TNKS2, AMOTL1 (angiomotin-like 1), and ZNRD2/SSSCA1 (Sjögren syndrome/scleroderma autoantigen 1); Fig. 7E], biological processes that are known to be functionally coupled to ADPr signaling and the previously reported functions of PARP14 (*25*, *34*). Together, our MS-based methodology and results will enable us to dissect the various functional roles in which PARP14 and its MD1 ADPr-hydrolase domain are involved.

### Macrodomain 1 of PARP14 and PARP9 regulate different cellular activities

**Fig. 8.**
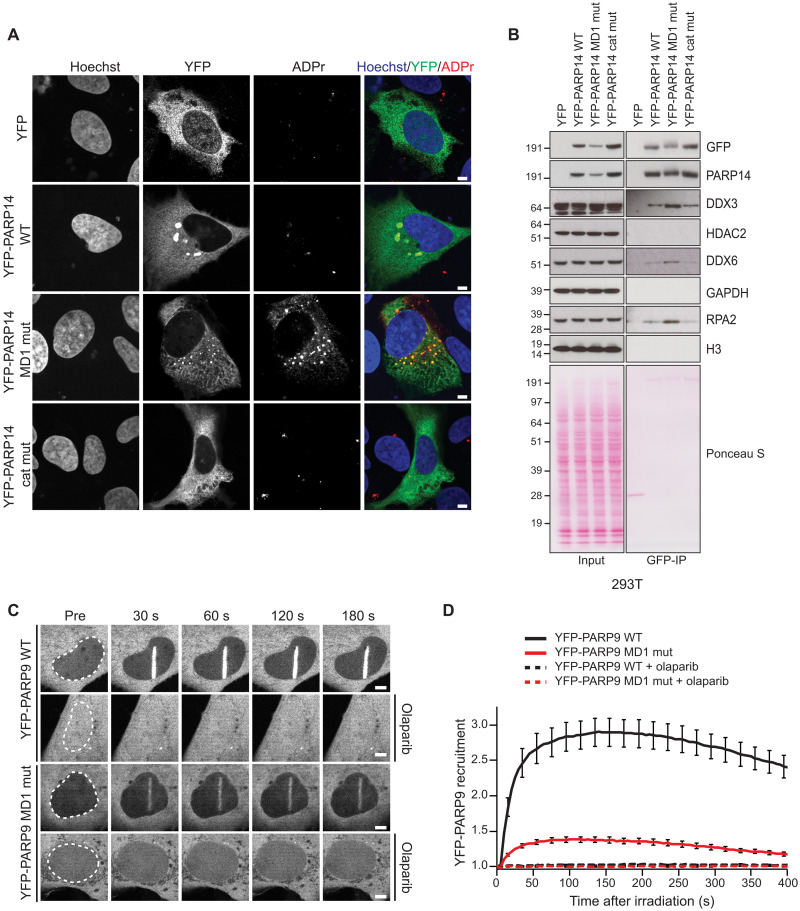
PARP14 and PARP9 macrodomain 1 regulates various cellular functions. (**A**) Confocal images of U2OS cells expressing YFP or YFP-PARP14 WT, MD1 mutant (MD1 mut), or catalytically inactive mutant (cat mut) stained with Hoechst (blue) and YFP (green) and for ADPr (poly/mono) (red). Scale bars, 5 μm. (**B**) 293T cells were cotransfected with the indicated YFP plasmid. Cell lysates and GFP-IPs were examined by Western blotting using the indicated antibodies. Ponceau S staining was used to indicate equal loading. (**C**) Confocal images and (**D**) recruitment kinetics of YFP-PARP9 and YFP-PARP9 macrodomain 1 mutant (MD1 mut) to sites of laser irradiation in the absence or presence of 1 μM olaparib. Scale bars, 5 μm.

Next, we aimed to investigate the role of PARP14 MD1 in a cellular context. First, we compared the localization of YFP-tagged PARP14 and its mutants expressed in U2OS cells. Here, we saw that YFP-PARP14 would localize primarily to the cytoplasm, that PARP14 WT would tend to occasionally form large foci around the periphery of the nucleus, and that these showed weak positive staining for ADPr (Fig. 8A). This localization was dependent on the catalytic activity of PARP14, as expression of catalytically inactive PARP14 resulted in no foci formation. Notably, the foci seen upon expression the YFP-PARP14 MD1 mutant were smaller, numerous, and stained strongly for ADPr (Fig. 8A). This result suggests the interplay between PARP14 ART activity and PARP14 MD1 hydrolysis activity modulate the cellular localization of PARP14.

We also explored whether PARP14 MD1 would also regulate protein interactions. We performed GFP-immunoprecipitation experiments using 293T cells overexpressing PARP14 WT, MD1 mutant, and the catalytically inactive mutant. We tested for the interaction with DEAD-Box Helcase 6 (DDX6), an RNA helicase that plays important roles in processing bodies (P-bodies) and in the immune response to viruses, for example, SARS2 (*57*), and has been shown to colocalize with PARP14 (*55*). Here, we saw that mutation of PARP14 MD1 resulted in a stronger interaction with DDX6 (Fig. 8B). Moreover, we observed a strong colocalization between DDX6 and YFP-PARP14 MD1 mutant by immunofluorescence and that this also colocalized with strong ADPr signal (fig. S5A). A stronger interaction with the PARP14 MD1 mutant compared to WT was also observed for DDX3, another P-body element (Fig. 8B). PARP14 has also been identified as an interactor of the DNA binding DNA repair/replication factor RPA using an MS pull-down approach (*58*). We observe an increased interaction between PARP14 and RPA when MD1 is mutated (Fig. 8B). On the other hand, HDAC2, which has been suggested to interact with PARP14 (*31*), did not show stable interaction with PARP14, regardless of MD1 activity (Fig. 8B).

PARP14 has been suggested to modulate STAT1 signaling and, therefore, modulate IFN-β production (*35*). To test the role of the catalytic activity or the hydrolytic activity of PARP14 MD1 on *IFN*β expression, we expressed YFP-PARP14 WT, MD1 mutant, or catalytic mutant and used quantitative polymerase chain reaction (qPCR) to examine the changes to *IFN*β after poly(I:C) treatment, which has been previously shown to induce *IFN*β expression (*59*). We found that expression of PARP14 catalytic mutant did not alter *IFN*β expression compared to YFP alone, while both PARP14 WT and the MD1 mutant dampened *IFN*β expression (fig. S5B).

Last, we looked at the model of PARP9 and examined the role of its macrodomain 1 (MD1). It has been previously shown that PARP9, as well as its binding partner DTX3L, recruits to sites of DNA damage and will modify proteins at DNA breaks (*42*, *43*). Here, we examined the recruitment of YFP-PARP9 WT and the MD1 mutant to sites of laser microirradiation. While we observed a robust recruitment of PARP9 WT to sites of damage, mutation of PARP9 MD1 resulted in a strong impairment of recruitment to sites of DNA damage (Fig. 8C). Furthermore, the recruitment of both WT and MD1 mutant was completely abolished upon treatment with the PARP1/2 inhibitor, olaparib, suggesting that the recruitment of PARP9 to sites of damage is through direct binding to ADP-ribose. Together, our results highlight the complexity of both ART and ADP-ribosyl hydrolase activity of PARP14 and PARP9 in different cellular functions.

## DISCUSSION

PARP14 is involved in the regulation of several cellular processes including DNA repair, immune and antiviral response, and RNA stability (*25*, *26*, *30*, *31*, *34*, *35*, *47*, *51*, *60*). However, it is still largely unclear how PARP14 activity is achieved and regulated. While PARP14 macrodomain 2 and 3 have been identified as binders of mono-ADPr substrates (*61*), the function of the first macrodomain has not yet been characterized. Our biochemical and cellular data along with recent data from others demonstrate that the PARP14 MD1 has hydrolytic activity and is a major cellular enzyme that controls the levels of PARP14 ADPr (*62*, *63*). This represents a rare example of an ART that also has ADP-ribosyl hydrolase activity. Analogously, PARP9 MD1 also has ADP-ribosyl hydrolase activity, but PARP9 lacks the transferase activity (*3*). PARP9 MD1 may contribute to the control of PARP14 ADPr levels, but it could equally control the ADPr levels of other transferases or under specific cellular conditions. However, the fact that PARP9 and PARP14 appear to be in the same complex and that they are expressed from the same genomic region (chromosome 3q21.1) suggests that the activities of these two PARPs regulate the same pathways (*35*, *47*). Together, our findings reveal two additional ADP-ribosyl hydrolase enzymes that are present in humans and many higher organisms in addition to the already known six ADP-hydrolases: PARG, ARH1/3, MacroD1/2, and TARG1 (*11*). Our discovery that PARP14 MD1 and PARP9 MD1 are specific for glutamate ADPr may suggest some redundancy with the other glutamate-ADPr targeting enzymes TARG1, MacroD1, and MacroD2 (*48*, *49*).

PARP14 MD1 and PARP9 MD1 are the most closely related human enzyme to SARS2 Nsp3 Mac1, more closely than to the human paralogs MacroD1 and TARG1 (Fig. 1). It is conceivable that coronaviruses and some other viruses bearing macrodomains such as alphaviruses and hepatitis E (*37*, *64*) hijacked MD1 at some point in evolution and now use it to oppose PARP14 ADPr antiviral activities. However, it cannot be ruled out that the specificities of these two macrodomains, at least on some ADPr sites in macromolecules, are different and may have diverged through evolution in the host-virus arms race. Linking to this, both PARP14 and other antiviral PARPs and viral macrodomains are under positive natural selection (*28*, *38*). Recent genetic data convincingly shows that PARP14 functions as an antiviral enzyme in a murine coronavirus model and that Mac1 counteracts this activity, antagonizing the interferon response and enabling viral replication (*25*, *36*).

PARP14 has been shown to play important roles in immunity and replication stress (*34*, *51*). We have used our published MS approach to unbiasedly identify the PARP14 modification protein targets (*56*). In particular, we identified a subset of ADP-ribosylated targets that are reversed via PARP14 MD1 hydrolase activity (Fig. 7). Fittingly, our results show that a majority of the identified proteins are involved in immunity, for example, PARP13, RNF114, and RNF166, which fits the finding that this domain is highly homologous to the viral SARS2 Mac1 (Fig 1). Many of the targets are also associated with the DNA damage response such as PARP1, XRCC1, and RPA1. Our data also confirms that PARP14 can reversibly ADP-ribosylate PARP13 (Figs. 6 and 7C). Although not catalytically active, PARP13 has been implicated in inhibiting the replication of multiple classes of viruses including retroviruses (*65*), alphaviruses (*23*), flaviviruses, and filoviruses (*27*). It is tempting to speculate that PARP14-induced ADPr of PARP13 might be involved in the regulation of PARP13 activity; namely, increased ADPr of PARP13 might affect its stability and/or binding affinity. However, further studies are required to understand the cross-talk between these two antiviral PARPs.

Furthermore, several PARP14 ADPr hits from our MS analysis were TNKS PARPs or their targets such as AMOTL1 and ZNRD2/SSSCA1 (Fig. 7). TNKS1 and TNKS2, both on the list of PARP14 MD1–regulated proteins, have been reported to control a large variety of pathways (*45*) including inhibiting the innate antiviral response by ADP-ribosylating virus-induced signal adaptor/ mitochondrial antiviral signaling protein, which facilitates the recruitment of the E3 ligase RNF146 for subsequent ubiquitination and degradation (*66*).

One of the major effects of macrodomain 1 from PARP14 that we have been able to identify is the change in cellular localization. Ectopic expression of PARP14 with active ART and MD1 domains results in large cellular bodies with unknown function in the cytoplasm, which show low levels of colocalization with ADPr (Fig. 8A). Loss of PARP14 ART activity resulted in loss of foci formation and loss of ADPr signal. Mutation of the MD1 ADP-hydrolase domain resulted in the formation of numerous smaller foci with strong staining with ADPr. Given that viral replication can depend on the hijacking of cellular vesicles, it would be of interest to identify what pathways or vesicles are altered with the changes to PARP14 activities. Our data does suggest a possible link to P-bodies with an increase interaction and colocalization with DDX6, an RNA helicase found in P-bodies (Fig. 8B and fig. S5A) (*57*); however, future studies will be needed to confirm this link. The localization of PARP9 protein is also dependent on its macrodomain 1. Specifically, we demonstrate that mutation of macrodomain 1 from PARP9 affects its ability to stably associate with sites of DNA damage (Fig. 8C).

Our data demonstrate that PARP14 is one of the major mono-ARTs in IFN-stimulated cells (fig. S3E). PARP14 has been shown to modulate both STAT1 phosphorylation and *IFN*β transcription (*25*, *47*). In poly(I:C)-stimulated 293T cells, we observe that PARP14 overexpression leads to lower levels of *IFN*β transcription, but we further demonstrate that this effect is dependent on PARP14 catalytic activity but not MD1 hydrolytic activity. The decrease in *IFN*β transcription may be due to lower levels of STAT1 phosphorylation, as PARP14 has been reported to block STAT1 phosphorylation (*47*). However, in another model (unstimulated delayed brain tumor cells), PARP14 overexpression led to an increase in *IFN*β expression, suggesting that the effect of PARP14 can vary between different cell models and types of stimulation (*25*).

PARP14 has been associated with the development of inflammatory diseases such as allergic asthma (*31*) and inflammatory arterial diseases (*47*) and various types of cancer including B cell lymphoma (*67*), prostate cancer (*68*), and hepatocellular carcinoma (*69*). Therefore, PARP14 has emerged as a potential drug target prompting the development of several PARP14 inhibitors (*39*, *70*), although none targeting PARP14 macrodomain 1. The discovery of the hydrolytic activity of PARP14, as well as PARP9, macrodomain 1 potentially presents a druggable target, together with SARS2 Nsp3 Mac1, that could be used to manipulate PARP9- and PARP14-dependent pathways or function as potent antivirals.

Together, our data identify PARP14 as a complex protein with the specific domains that enables it to function as a writer (ART), reader (MD2 and MD3) (*61*), and eraser (MD1) of ADPr in addition to nucleic acid binding domains (Fig. 1A). This, together with the interplay of the PARP9/DTX3L complex and ubiquitylation signaling (*44*, *47*), is expected to have far-reaching consequences on the physiology of the cell and human disease.

## MATERIALS AND METHODS

### Hydrolase activity analysis using luminescence detection of ADPr

The ADP-ribosylated peptides were chemically synthesized (table S1). Serine-ADPr and arginine-ADPr peptides were synthesized as previously described (*71*, *72*). Chemical synthesis of the glutamate-ADPr peptide was previously described (*73*). The hydrolytic assay against the ADP-ribosylated peptides was performed as previously described (*74*). Briefly, 10 μM substrate peptides (arginine-ADPr, serine-ADPr, or glutamate-ADPr) was hydrolyzed using 1 μM PARP14 MD1, PARP14 MD1mut, PARP14 MD2, PARP14 MD3, PARP9 MD1, PARP9 MD2, or S2 Mac1. ARH1, ARH3, and MacroD1 served as positive controls for arginine-ADPr, serine-ADPr, and glutamate-ADPr, respectively. Hydrolysis was carried out for 1 hour at 30°C in assay buffer [50 mM tris-HCl (pH 7.5), 200 mM NaCl, 10 mM MgCl_2_, 1 mM dithiothreitol (DTT), and 0.2 μM Nudix hydrolase 5 (NUDT5) for arginine-ADPr and serine-ADPr and 50 mM Pipes (pH 6.9), 200 mM NaCl, 10 mM MgCl_2_, 1 mM DTT, and 0.2 μM NUDT5 for glutamate-ADPr]. The reactions were analyzed using the AMP-Glo assay kit (Promega) following the manufacturer’s recommendations. Luminescence was read using a SpectraMax M5 plate reader with the SoftMax Pro software (Molecular Devices). Data were analyzed using GraphPad Prism.

### In vitro protein (ADP-ribosyl) hydrolase assay

PARP14 WWE-CAT (1 μM) with or without PARP14 MD3 (2 μM) was incubated with 50 μM NAD^+^ (spiked with ^32^P NAD^+^) in reaction buffer [50 mM tris-HCl (pH 8.0), 100 mM NaCl, and 2 mM MgCl_2_]. Reactions were incubated at 37°C for 3 hours, then stopped by the addition of 0.5 μM PARP14i, and further passed through a preequilibrated G-25 column to remove excess of NAD^+^. The flow-through was ADP-ribosylated PARP14 WWE-CAT and PARP14 MD3, which were used for hydrolase assays. Next, ADP-ribosylated substrates were incubated with PARP14 and PARP9 macrodomains (2 μM) for 1 hour. The reactions were subsequently stopped by the addition of 4× LDS sample buffer (Life Technologies) and incubation at 95°C for 5 min. Samples were then analyzed by SDS–polyacrylamide gel electrophoresis (SDS-PAGE) and autoradiography.

### In vitro DNA and RNA (ADP-ribosyl) hydrolase assay

DNA and RNA (ADP-ribosyl) hydrolase assays were carried out as described previously (*21*). All buffers were prepared using deoxyribonuclease/ribonuclease (RNase)–free water and filter-sterilized before use. Briefly, 0.25 μM Cy3–labeled RNA or DNA (table S2) was mixed with 500 μM NAD^+^ and 2 μM PARP14 WWE-CAT. Reactions were incubated for 1 hour at 30°C. The ADPr reaction was terminated by the addition of 0.1 μM PARP14i; the reaction products were not purified before the addition of the macrodomains unless stated specifically (see the purification procedure below). Hydrolysis of the ADP-ribosylated DNA or RNA was initiated by the addition of 4 μM macrodomains from PARP14, PARP9, or SARS2 Mac1, followed by incubation of the reactions for 30 min at 30°C. Hydrolysis was stopped by adding proteinase K (50 ng/μl) and 0.15% SDS, followed by incubation for 30 min at 50°C. Tris-borate EDTA urea sample buffer [2×; 8 M urea, 20 μM EDTA (pH 8.0), 2 μM tris-HCl (pH 7.5), and bromophenol blue] was subsequently added, and the samples were incubated at 95°C for 3 min. The samples were run on a prerun denaturing urea PAGE gel [20% (w/v) polyacrylamide, 8 M urea, and 1× TBE] at 7 W per gel in 0.5× TBE. The gels were visualized with laser excitation for Cy3 at 532 nM using a PharosFX Molecular Imager (Bio-Rad).

In fig. S1B, purified ADP-ribosylated ssDNA was generated and used as a substrate for the hydrolysis assay. Here, 50 μM ssDNA were incubated with 20 μM PARP14 WWE-CAT and 10 mM NAD^+^ in reaction buffer [20 mM Hepes-KOH (pH 7.6), 5 mM MgCl_2_, and 1 mM DTT]. The reactions were incubated at 37°C for 3 hours and stopped by adding proteinase K (50 ng/μl) and 0.15% SDS, followed by incubation at 50°C for 30 min. Then, the reaction mixture was incubated at 95°C for 5 min to inactivate proteinase K. The reaction was further passed onto a preequilibrated G-25 column to remove the excess NAD^+^.

For dsDNA annealing, 5 μM Cy3-labeled 5′ phosphorylated or 3′ phosphorylated E21 ssDNA was annealed with 10 μM various nonphosphorylated oligonucleotides (1:2 molar ratio) to create blunt, gapped, nicked, overhang, and forked dsDNA (fig. S2). The oligonucleotides were mixed in annealing buffer [10 mM tris (pH 8.0), 50 mM NaCl, and 0.5 mM EDTA] and incubated at 95°C for 5 min, followed by gradual cooling to 25°C over 1 hour. The samples were run on native 20% acrylamide gel to check for annealing completion.

For dsDNA modification reaction, 0.25 μM annealed dsDNA was mixed with 5 μM PARP14-WWE-Cat and 1 mM NAD^+^ in reaction buffer [20 mM Hepes (pH 7.5), 50 mM KCl, 5 mM MgCl_2_, and 1 mM DTT] at 37°C for 90 min. PARP14 inhibitor (0.5 μM) was added to stop the reactions. For macrodomain treatment, 4 μM PARP14-MD1 was added to the reaction mixture and incubated at 37°C for 60 min. Samples were incubated with proteinase K (50 ng/μl) and 0.15% SDS at 37°C for 30 min, mixed with 2× TBE-urea loading buffer, and incubated at 95°C for 3 min. Subsequently, samples were run on a 20% acrylamide TBE-urea gel. The gels were visualized with laser excitation for Cy3 at 532 nM using a PharosFX Molecular Imager (Bio-Rad).

### Competitive hydrolase assay

The purified ADP-ribosylated ssDNA was generated as described above. The reaction was further passed onto preequilibrated G-25 column to get rid of the excess of NAD^+^. The flow-through was the mixture of modified and unmodified ssDNA and used in the competitive hydrolase assay.

ADP-ribosylated PARP14 WWE-CAT (0.5 μM) were incubated with or without PARP14 MD1 (2 μM) in the presence or absence of ADP-ribosylated ssDNA (4 μM) in reaction buffer [20 mM Hepes-KOH (pH 7.6), 5 mM MgCl_2_, and 1 mM DTT] at 37°C for indicated times. The reactions were subsequently stopped by the addition of 4× LDS sample buffer (Life Technologies) and incubation at 95°C for 5 min. Samples were then analyzed by SDS-PAGE and autoradiography.

### Plasmids and mutagenesis

Full-length PARP14 cloning was performed by Gateway cloning (Invitrogen) according to the manufacturer’s instructions. PARP14-encoding pEZ-M11 mammalian expression vector (300 ng) obtained from GeneCopoeia was directly set up for BP recombination reaction with pDONR221 vector without PARP14 insert amplification. The reaction was stopped by adding 1 μl proteinase K (Invitrogen) and after incubation for 10 min at 37°C. Competent Stable *E. coli* (New England Biolabs) was transformed with 2 μl of the BP reaction mix. For transfer into the destination vector, 100 ng of positive pENTR clone DNA was incubated with 100 ng of pDEST-N-YFP/FRT/TO pcDNA5 and LR Clonase enzyme mix for 2 hours at room temperature. Plasmid DNA was isolated for positive clones and verified by Sanger sequencing. PARP9 was cloned into of pDEST-N-YFP/FRT/TO pcDNA5 using gateway cloning.

PARP14 and PARP9 macrodomains were cloned into a pNIC28-Bsa4 vector, which adds an N-terminal His_6_-TEV cleavage site to the proteins to aid protein purification. PARP14 and PARP9 point mutations were introduced through site-directed mutagenesis PCR using the QuikChange Lightning kit (Agilent) or the Q5 Site-Directed Mutagenesis Kit (New England Biolabs) with primers described in table S3 and confirmed by Sanger sequencing.

Mammalian expression vectors encoding FLAG-MacroD1, FLAG-MacroD2, FLAG-PARG, FLAG-SARS2 Mac1, and GFP-PARP13 were generated by gateway cloning as described previously (*54*). PaTagRFP-H2B (histone 2B) (*75*) and GFP-DarT2 (*Thermus aquaticus*) (*76*) were previously described.

### Protein expression and purification

BL21(DE3)-R3-pRARE cells were transformed with PARP14 and PARP9 macrodomain encoding constructs and grown at 37°C in LB medium supplemented with appropriate antibiotics until optical density at 600 nm (OD_600_) 0.5 to 0.6, then cooled to 18°C, and supplemented with 0.5 mM isopropyl-β-d-thiogalactopyranoside at an OD_600_ of 0.8 to induce protein expression overnight. Cells were harvested by centrifugation, resuspended in lysis buffer [50 mM Hepes (pH 7.5), 500 mM NaCl, 20 mM imidazole, 5% glycerol, 0.5 mM tris(2-carboxyethyl)phosphine (TCEP), and 1:2000 Calbiochem protease inhibitor cocktail set III), and lysed by sonication. Proteins were purified by Ni^2+^–nitrilotriacetic acid chromatography (Jena Bioscience) and eluted stepwise in binding buffer with 40 to 250 mM imidazole. Proteins were further purified by size exclusion chromatography (Superdex 75, GE HealthCare) in a buffer consisting of 50 mM Hepes (pH 7.5), 300 mM NaCl, 5% glycerol, and 0.5 mM TCEP. PARP14 MD1 was additionally purified by ion exchange chromatography using a HiTrap SP HP (5 ml; GE HealthCare) equilibrated in 25 mM Hepes (pH 7.5), 75 mM NaCl, and 0.5 mM TCEP. The purity of protein preparations was assessed using SDS-PAGE and Coomassie Brilliant Blue staining, and aliquots were stored at −80°C until use.

### Cell culture

Human U2OS osteosarcoma [American Type Culture Collection (ATCC), HTB-96], embryonic kidney 293T (ATCC, CRL-3216), and A549 (ATCC, CCl-185) cell lines were purchased from ATCC. Cells were grown in Dulbecco’s modified Eagle’s medium (Sigma-Aldrich) supplemented with 10% fetal bovine serum (FBS; Gibco) and penicillin-streptomycin (100 U/ml; Gibco). All cell lines were cultured in a humidified atmosphere at 37°C with 5% CO_2_. Human embryonic kidney 293T (293T) and U2OS cells were plated in 10-cm dishes 24 hours before cells were transfected with the indicated plasmids. 293T cells were transfected using PolyFect (QIAGEN), while U2OS cells were transfected using TransIT-LT1 Transfection Reagent (Mirus Bio), according to the manufacturer’s protocol. Cells were treated with DMSO, 0.5 μM PARP14i (RBN012759, MedChemExpress), 5 μM PARGi (PDD00017273, Sigma-Aldrich), or IFN-γ (100 ng/ml; Merck) for 24 hours.

### Immunoprecipitation

293T and U2OS cells were collected 24 or 48 hours after transfection and washed two times with phosphate-buffered saline (PBS). Cells were lysed with Triton X-100 lysis buffer [50 mM tris-HCl (pH 8.0), 100 mM NaCl, and 1% Triton X-100] supplemented with 5 mM MgCl_2_, 0.1% Benzonase (Sigma-Aldrich), protease and phosphatase inhibitors (Roche), olaparib (Cayman Chemical; 1 μM for U2OS and 2 μM for 293T cells), and 1 μM PARGi PDD00017273 (Sigma-Aldrich) for 30 min at 4°C. Protein concentrations were determined by Bradford Protein Assay (Bio-Rad) and normalized for equal protein amounts. Cell lysates were incubated with GFP-Trap magnetic agarose beads (ChromoTek) on an orbital rotator for 2 hours at 4°C. Beads were pelleted using a magnetic separation rack and washed five times with Triton X-100 lysis buffer [50 mM tris-HCl (pH 8.0), 800 mM NaCl, and 1% Triton X-100]. Proteins were eluted with 2× NuPAGE LDS sample buffer (Invitrogen) supplemented with DTT (Sigma-Aldrich), boiled for 5 min at 95°C, and analyzed by Western blotting.

### Dot blot

For genomic DNA and total RNA extraction, 1.2 × 10^6^ of U2OS WT cells were seeded on a 10-cm dish. Cells were transfected using TransIT-LT1 (Mirus, MIR2300) according to the manufacturer’s instructions. TARG1 KO cells were transfected with GFP-DarT2 was used as a positive control for DNA-ADPr (*76*). Cells were harvested in ice-cold PBS 24 hours after transfection and pelleted at 300*g* for 5 min in a benchtop centrifuge.

Genomic DNA was extracted using the DNeasy Blood & Tissue Kit (QIAGEN) according to the manufacturer’s instructions with the addition of RNA digestion with RNase A (10 μg/ml; Invitrogen, 8003089) for 5 min at room temperature before the addition of proteinase K. DNA concentration was measured using a spectrophotometer (DeNovix, DS-11 FX), and the concentration was normalized across the samples. Total RNA was extracted from cells using the RNeasy Mini Kit (QIAGEN) according to the manufacturer’s instructions. RNA concentration was measured using a spectrophotometer, and the concentration was normalized across the samples using RNase-free water (Invitrogen).

Equal amounts, 100 ng, of nucleic acids were dotted onto a nitrocellulose membrane (0.45 μm; Amersham Protran) and cross-linked using 1200 J at 254 nm with an ultraviolet cross-linker (Starlinker) (*19*, *76*). The membranes were blocked with blocking buffer [5% nonfat dried milk (w/v) in PBST] for 1 hour before the addition of primary antibodies diluted in blocking buffer for 1 hour at room temperature (table S4). The membranes were washed three times with PBST and then incubated with HRP-conjugated secondary antibodies diluted in blocking buffer for 1 hour at room temperature. Blots were washed three times in PBST. Chemiluminescence was detected using the SuperSignal West Dura Extended Duration Substrate (Thermo Fisher Scientific) on Hyperfilm ECL film (Cytiva).

The RhsP2-positive control was created using RhsP2 protein provided by J. Whitney (*77*). The RNA-ADPr was synthesized by incubating double-stranded RNA oligonucleotide with 1 μM RhsP2 and 1 mM NAD^+^ in 20 mM Hepes-KOH (pH 7.6), 50 mM KCl, 5 mM MgCl_2_, and 1 mM DTT buffer for 1 hour at 37°C.

### Western blotting

Cells were lysed, and protein concentration was measured as described above. Proteins were boiled in 1× NuPAGE LDS sample buffer (Invitrogen) with 60 mM DTT (Sigma-Aldrich) and resolved on NuPAGE Novex 4 to 12% bis-tris gels (Invitrogen) in 1× NuPAGE MOPS SDS Running Buffer (Invitrogen) at 150 V. Proteins were transferred onto nitrocellulose membranes (Bio-Rad) using Trans-Blot Turbo Transfer System (Bio-Rad). The membranes were stained with Ponceau S Staining Solution (Thermo Fisher Scientific) to check the transfer quality, rinsed with water, and blocked in 5% (w/v) nonfat dried milk in PBS buffer with 0.1% (v/v) Tween 20 (PBST) for 1 hour at room temperature. This was followed by overnight incubation with primary antibody as indicated in table S4 at 4°C. The next day, membranes were washed in PBST and incubated with HRP-conjugated antibodies at room temperature for 1 hour. Membranes were visualized on Hyperfilm ECL films (Cytiva) after adding Pierce ECL Western Blotting Substrate (Thermo Fisher Scientific).

### Sequence and phylogenetic analysis

For multiple-sequence alignments, Jalview v2 (*78*) and MAFFT7 (*79*) were used. The phylogenetic tree of macrodomains was generated with SplitsTree4 (v4.15.1) (*80*) using the neighbor-joining method and confidence levels estimated using 1000 cycles of the bootstrap method. Pairwise identities were determined using the Needleman-Wunsch algorithm implemented as part of the European Molecular Biology Laboratory–European Bioinformatics Institute search and sequence analysis server (*81*). Structural alignments and analyses, as well as figure preparation, were carried out using PyMOL (Molecular Graphics System, version 2.3.3; Schrӧdinger LLC). PARP domain architecture was visualized using IBS illustrator (*82*).

### Reverse transcription qPCR

Following procedures previously described in (*83*), total RNAs from 293T cells were purified with the RNeasy Plus Micro Kit (QIAGEN), and then, 0.5 μg of total RNA was used for cDNA synthesis with QuantiTect Reverse Transcription Kit according to the manufacturer’s instructions. The cDNAs were detected by quantitative real-time PCR using the Rotor-Gene SYBR Green PCR Kit and the Rotor-Gene Q (QIAGEN). Primer pairs for reverse transcription qPCR are given in table S5. The relative gene expression analysis of *INF*β was performed using the ddCt method normalized to hypoxanthine phosphoribosyltransferase 1 (*HPRT1*).

### Enrichment of ADPr proteins for MS analysis

293T cells were cultured as five technical replicates in a humidified incubator at 37°C with 5% CO_2_ in 15-cm dishes. The cells were transfected with plasmids encoding either YFP-PARP14 WT or YFP-PARP14 MD1 mutant sequences as described above. Cells were gently washed with PBS 24 hours after transfection and then immediately lysed in radioimmunoprecipitation assay (RIPA) buffer [50 mM tris-HCl (pH 8.0), 100 mM NaCl, and 1% Triton X-100] supplemented with 5 mM MgCl_2_, 0.1% Benzonase (Sigma-Aldrich), protease and phosphatase inhibitors (Roche), 2 μM olaparib (Cayman Chemical), and 1 μM PARGi PDD00017273 (Sigma-Aldrich) for 30 min at 4°C.

Lysates were split evenly across either Af1521-linked glutathione Sepharose 4B beads or empty control beads (Cytiva). The purification of the GST-Af1521 macrodomain was performed essentially as described previously (*56*, *84*). The beads were incubated with the lysates for 8 hours at 4°C tumbling end over end to facilitate binding between the ADP-ribosylated proteins and the Af1521-containing beads. After binding, the beads were transferred to new tubes three times and were washed two times after each tube change in RIPA buffer without supplements. Proteins were digested on beads with RIPA buffer supplemented with 500 ng of trypsin (sequencing grade; Promega) per sample and incubated overnight at 30°C with mild shaking. To separate the peptides from the beads, samples were spun through a 0.45 μM filter (Ultrafree-MC, Millipore), and then reduced, and alkylated with 5 mM TCEP and 5 mM chloroacetamide for 30 min at room temperature.

Following procedures previously described in (*85*), peptides were subjected to StageTip clean-up by desalting and purifying them on C18 discs at high pH. Briefly, quad-layer StageTips were prepared using four punch-outs of C18 material (C18, 47 mm; Sigma-Aldrich, Empore SPE Disks). StageTips were equilibrated using 100 μl of methanol, 100 μl of 80% acetonitrile (ACN) in 200 mM ammonium hydroxide, and 75 μl of 50 mM ammonium hydroxide two times. Samples were supplemented with one-tenth volume of 200 mM ammonium hydroxide (pH, >10), just before loading them on the StageTip. The StageTips were subsequently washed twice with 150 μl of 50 mM ammonium hydroxide and afterward eluted using 80 μl of 30% ACN in 50 mM ammonium hydroxide. All samples were dried to completion in Protein LoBind tubes (Eppendorf) using a SpeedVac for 2 hours at 60°C, after which the dried peptides were dissolved using 11 μl of 0.1% formic acid and stored at 20°C until MS analysis.

### MS analysis

Following procedures previously described in (*86*), MS samples were analyzed on an EASY-nLC 1200 system (Thermo Fisher Scientific) coupled to an Orbitrap Exploris 480 mass spectrometer (Thermo Fisher Scientific). Separation of peptides was performed using 20-cm columns (internal diameter, 75 μm) packed in-house with ReproSil-Pur 120 C18-AQ 1.9-μm beads (Dr. Maisch). Elution of peptides from the column was achieved using a gradient ranging from buffer A (0.1% formic acid) to buffer B (80% ACN in 0.1% formic acid) at a flow of 250 nl/min. The gradient length was 80 min per sample, including ramp-up and wash-out, with an analytical gradient of 60 min ranging from 5% B to 38% B. Analytical columns were heated to 40°C using a column oven, and ionization was achieved using a Nanospray Flex NG ion source. Spray voltage was set to 2 kV; ion transfer tube temperature was set to 275°C, and radio frequency funnel level was set to 40%. Full scan range was set to 300 to 1300 mass/charge ratio (*m*/*z*). MS1 resolution was set to 120,000. MS1 AGC target was set to “200” (2,000,000 charges), and MS1 maximum injection time was set to “Auto.” Precursors with charges 2 to 6 were selected for fragmentation using an isolation width of 1.3 *m*/*z* and fragmented using higher-energy collision disassociation with normalized collision energy of 25. Precursors were excluded from resequencing by setting a dynamic exclusion of 80 s. MS2 resolution was set to 30,000. MS2 AGC target was set to 200 (200,000 charges). Intensity threshold was set to 360,000 charges per second. MS2 maximum injection time was set to Auto, and TopN was set to 13.

### Data processing

All RAW files were analyzed using MaxQuant software (version 1.5.3.30) using default settings, except with label-free quantification enabled (*87*). The human FASTA database used in this study was downloaded from UniProt on 25 June 2023 (UP000005640). All data were filtered by posterior error probability to achieve a false discovery rate (FDR) of <1% (default) at both the peptide-spectrum match and the protein assignment levels.

### Data filtering and statistical analysis

Beyond automatic filtering and FDR correction applied by MaxQuant during data processing, data S1 and S2 were manually stringently filtered to ensure robust quantification of differential experimental groups using the freely available Perseus software (*88*). This filtering includes log_2_ transformations, *n* = 5 filtering within at least one group, filtering proteins identified with less than two peptides, column-wise imputation (down shift, 1.8; width, 0.3), two-sample *t* tests for differential expression, and enrichment analysis through FDR-controlled Fisher exact testing. Principal components analysis was performed using the R (version 4.3.1 “Beagle Scouts”) function prcomp, after transposing and *z* score normalizing the rows following the filters described above. Plots were generated using R and the “ggplots” package (version 3.4.2).

UniProt entries complete with Gene Ontology and keywords were downloaded concomitantly with the fasta file used to build the search space in MaxQuant, and these were mapped to the filtered dataset to perform gene set enrichment analysis. To this end, proteins that were significantly enriched in the Af1521 group over the control were assigned as the background dataset, while the proteins that were significantly up-regulated in the MD1 samples over the WT was considered foreground. The significantly enriched terms, found in data S3, was found by Fisher’s exact test with Benjamini-Hochberg FDR corrected *P* values >5%.

The online STRING database (version 11.5) was used for generation of protein interaction (*89*), and Cytoscape (version 3.10.0) was used for manual annotation and visualization of the STRING (*90*) together with the Omics Visualizer App (*91*).

### Data availability

The MS proteomics data have been deposited to the ProteomeXchange Consortium via the PRIDE (*92*) partner repository with the dataset identifier PXD043452.

### Microscopy

Following procedures previously described in (*83*), U2OS cells were plated on an eight-well μ-slide glass bottom chamber slide (ibidi) and transfected with an expression plasmid for YFP-PARP9 WT or MD1 mutant together with a plasmid for expression of photoactivable tagged H2B (PaTR-H2B) 48 hours before imaging. For cell sensitization before laser irradiation at 405 nm, growth medium was aspirated from the Lab-Tek and replaced with fresh medium containing Hoechst 33342 (0.3 μg/ml). Immediately before imaging, the Hoechst containing medium was replaced with imaging media [phenol red–free Leibovitz’s L-15 medium (Life Technologies) supplemented with 20% FBS, penicillin (100 μg/ml), and streptomycin (100 U/ml). Live-cell microscopy was carried out on an Olympus IX-83 inverted microscope equipped with a Yokogawa SoRa superresolution spinning-disk head, a UPlanAop 60x/1.5 numerical aperture oil-immersion objective lens and a Prime BSI scientific complementary metal-oxide semiconductor camera. The fluorescence of YFP and PaTagRFP-H2B was excited with 488-nm and 561-nm solid state lasers, respectively, and fluorescence detection was achieved with band-pass filters adapted to the fluorophore emission spectra. Laser microirradiation at 405 nm was performed along a 15-μm line through the nucleus for 250 ms using a single-point scanning head (Olympus cellFRAP) coupled to the epifluorescence backboard of the microscope. To ensure reproducibility, laser power at 405 nm was measured at the beginning of each experiment and set to 110 μW at the sample level. For time course experiments, images were collected every 5 s. For the live-cell imaging experiments, cells were maintained at 37°C with a heating chamber. Protein accumulation at sites of damage (*A_d_*) was then calculated as

$$A_{d}=\frac{I_{d}-I_{\mathrm{bg}}}{I_{n}-I_{\mathrm{bg}}}$$

The intensity within the microirradiated area was then normalized to the intensity before damage induction. Photoactivated H2B was used as a reference to indicate where irradiation had occurred.

For immunofluorescence, U2OS cells were plated on an eight-well μ-slide glass bottom chamber slide, while 293T cells were plated on poly-l-lysine–coated coverslips. Cells were transfected as described above. Cells were fixed 24 hours after transfection for 20 min at −20°C with ice-cold methanol:acetone (1:1). Cells were washed twice with PBS before being blocked for 60 min in blocking buffer (3% bovine serum albumin in PBS + 0.2% Tween). Cells were incubated in primary antibody overnight at 4°C before being washed three times with PBS + 0.1% Triton. Cells were incubated with secondary antibody in blocking buffer with Hoechst 33342 (1 μg/ml). Cells were washed three times with PBS + 0.1% Triton before being mounted on slides using MOWOIL (Merck) or being imaged directly. Immunofluorescence was carried on Olympus IX-83 inverted microscope as described above using 405-, 488-, and 633-nm solid-state lasers and with band-pass filters adapted to the fluorophore emission spectra.
