# Supplementary material for: PARP14 is a PARP with both ADP-ribosyl transferase and hydrolase activities
Source: Sci Adv. 2023 Sep 13;9(37):eadi2687. doi: 10.1126/sciadv.adi2687 (PMC10499325; doi:10.1126/sciadv.adi2687)
Supplement: Supplementary file 1 — Figs. S1 to S5 Tables S1 to S5 Legends for data S1 to S3 [file sciadv.adi2687_sm.pdf]

Science Advances
AAAS

Supplementary Materials for

PARP14 is a PARP with both ADP-ribosyl transferase and hydrolase activities

Nina Đukić *et al.*

Corresponding author: Rebecca Smith, rebecca.smith@path.ox.ac.uk; Ivan Ahel, ivan.ahel@path.ox.ac.uk

Sci. Adv. **9**, eadi2687 (2023)
DOI: 10.1126/sciadv.adi2687

The PDF file includes:

Figs. S1 to S5
Tables S1 to S5
Legends for data S1 to S3

Other Supplementary Material for this manuscript includes the following:

Data S1 to S3

Supplementary Figures:

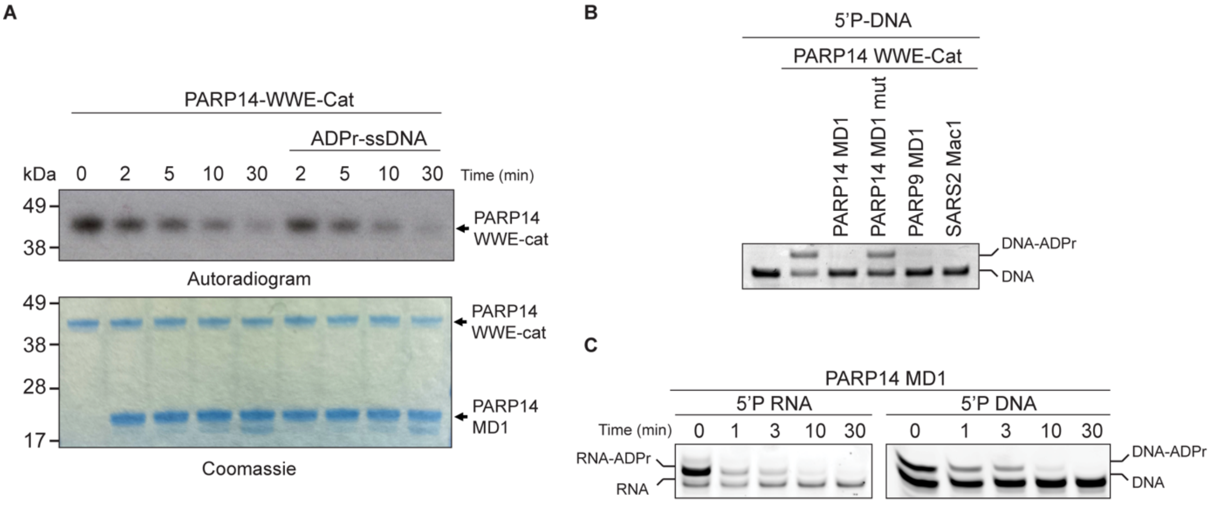

Supplementary Figure 1: PARP14 macrodomain 1 reverses protein, DNA and RNA ADP-ribosylation. (A) Time-course reversal of ADP-ribosylated PARP14 WWE-cat by PARP14 MD1 in the presence or absence of excess ADPr-ssDNA. ADP-ribosylated PARP14 WWE-cat was incubated with or without PARP14 MD1 in presence or absence of eight fold molar excess of ADP-ribosylated ssDNA for the indicated times. (B) PARP14 MD1 reverses ADP-ribosylation of 5'P-ssDNA. ssDNA with 5′ phosphate and 3′ Cy3 were ADP-ribosylated using PARP14 WWE-cat. Subsequently, the ADP-ribosylated DNA was purified and used as a substrate for de-ADP-ribosylation reactions with PARP14 MD1, MD1 mut, MD2, MD3 and SARS2 Mac1 (C) Time-course reversal of ADP-ribosylated 5′ phosphate ssRNA or 5′ phosphate ssDNA using PARP14 MD1. ADP-ribosylated 5' and 3' phosphorylated ssRNA and ssDNA were treated with PARP14 MD1 and samples were taken at the indicated timepoints.

5'P-dsDNA set

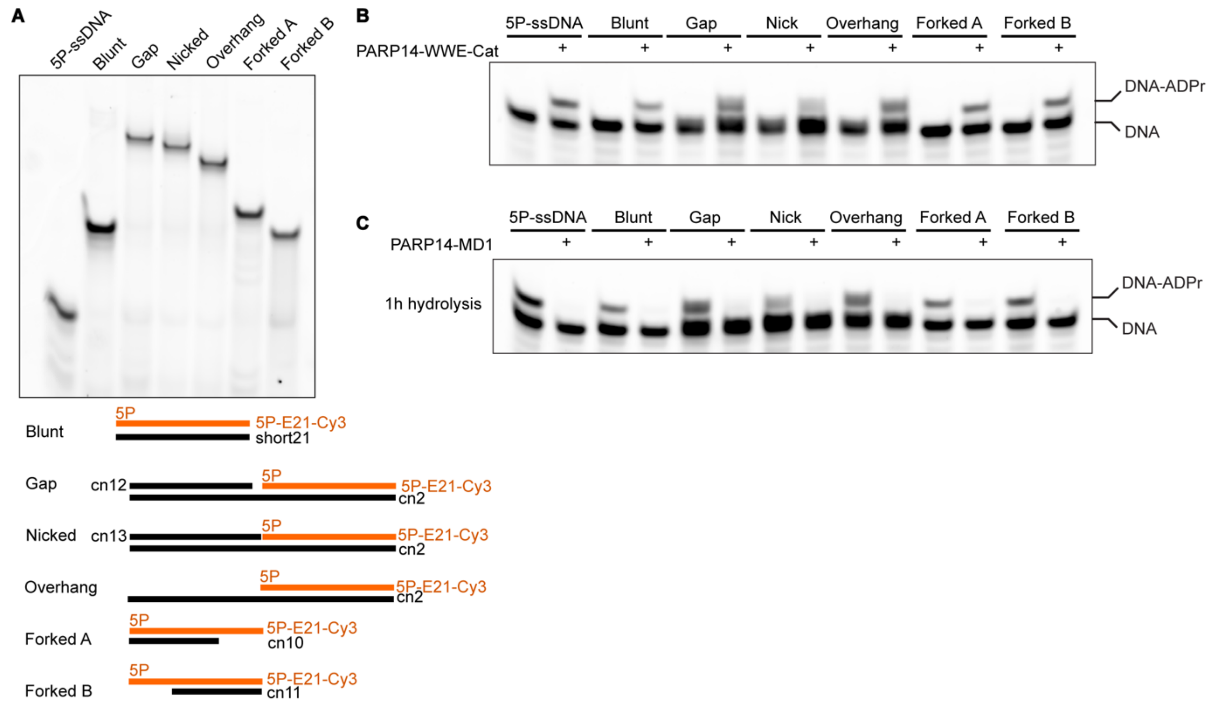

3'P-dsDNA set

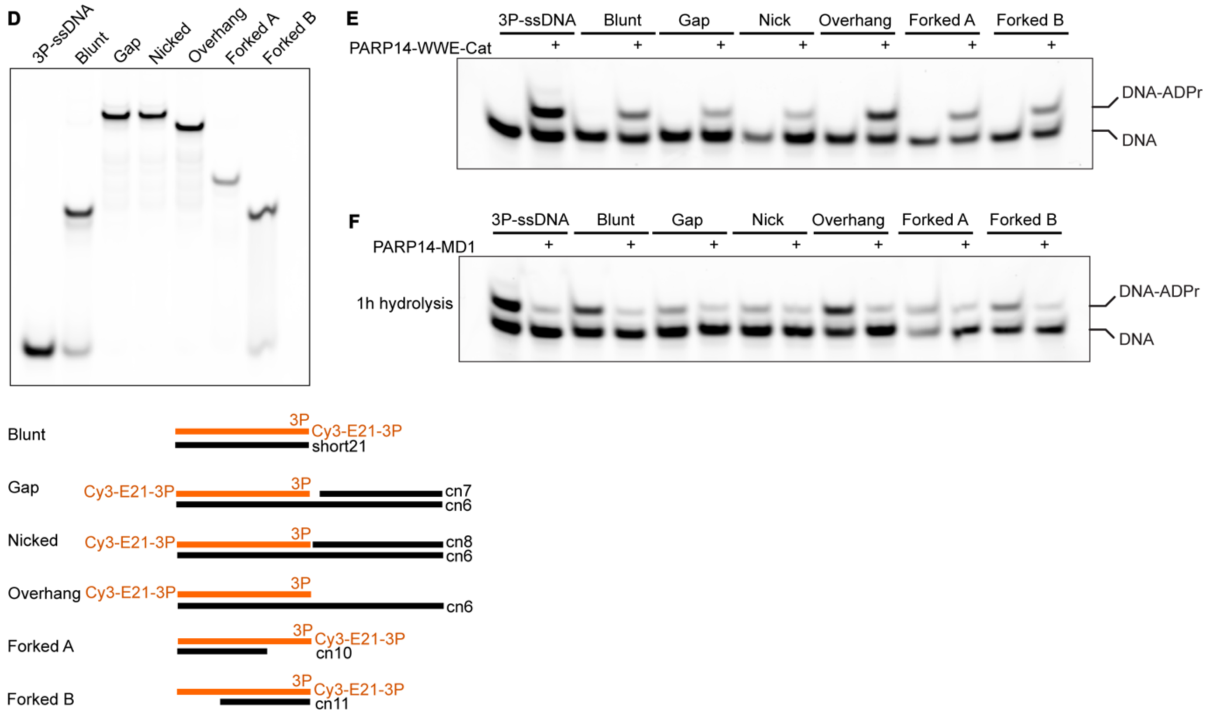

Supplementary Figure 2: PARP14 macrodomain 1 can remove ADPr from various dsDNA substrates. (A and D) Quality control of annealed 5'P-dsDNA and 3'P dsDNA analysed by native polyacrylamide gel electrophoresis. (B and E) Annealed 5'P-dsDNA and 3'P dsDNA were ADP-ribosylated with 5 µM PARP14 WWE-cat and analysed by urea polyacrylamide gel electrophoresis (C and F) ADP-ribosylated 5P-dsDNA from B and ADP-ribosylated 3'P dsDNA from E were treated with 4 µM PARP14 MD1 and analysed by urea polyacrylamide gel electrophoresis.

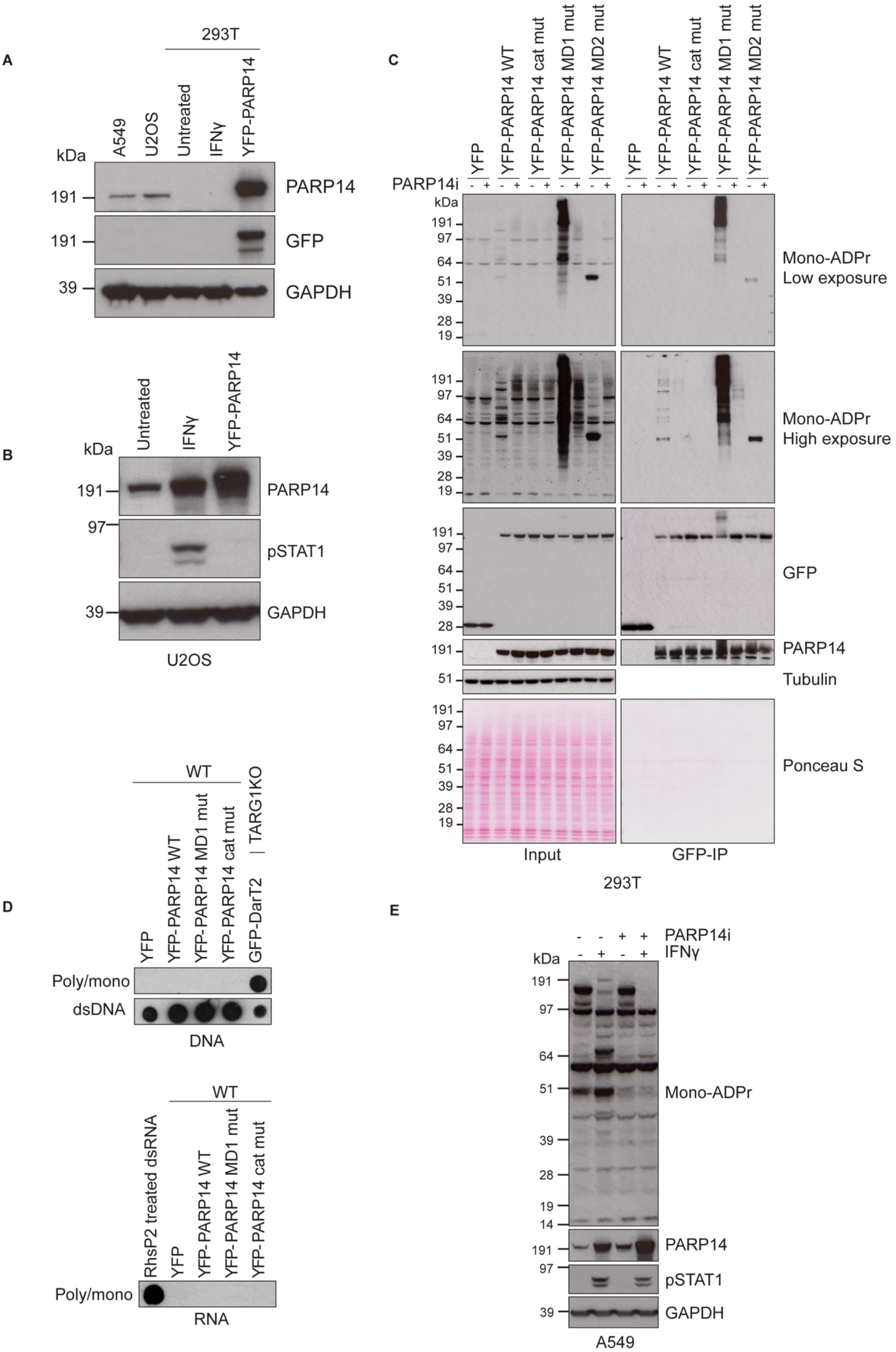
A
293T
A549
U2OS
Untreated
IFNγ
YFP-PARP14
kDa
191
191
39
PARP14
GFP
GAPDH
B
Untreated
IFNγ
YFP-PARP14
kDa
191
97
39
PARP14
pSTAT1
GAPDH
U2OS
C
YFP
YFP-PARP14 WT
YFP-PARP14 cat mut
YFP-PARP14 MD1 mut
YFP-PARP14 MD2 mut
PARP14i
kDa
191
97
64
51
39
28
19
Mono-ADPr Low exposure
Mono-ADPr High exposure
GFP
PARP14
Tubulin
Ponceau S
Input
GFP-IP
293T
D
WT
YFP
YFP-PARP14 WT
YFP-PARP14 MD1 mut
YFP-PARP14 cat mut
GFP-DarT2
TARG1KO
Poly/mono
dsDNA
DNA
RhsP2 treated dsRNA
Poly/mono
RNA
E
PARP14i
IFNγ
kDa
191
97
64
51
39
28
19
14
Mono-ADPr
PARP14
pSTAT1
GAPDH
A549

Supplementary Figure 3: PARP14 protein levels in cells and its activities on protein and nucleic acid substrates in cells. (A) A549, U2OS and 293T cells were examined for their PARP14 levels. 293T cells were stimulated with IFNγ (100 ng/mL) for 24 h or transfected with YFP-PARP14 WT. GFP was used to show expression of the YFP-tagged PARP14. GAPDH was used as a loading control. (B) Levels of overexpressed PARP14 are similar to levels of endogenous PARP14 after induction with IFNγ in U2OS cells. U2OS cells were stimulated with IFNγ (100 ng/mL) for 24 h or transfected with YFP-PARP14 WT. Phosopho-STAT1 was used to demonstrate IFNγ stimulation. GAPDH was used as a loading control. (C) PARP14 is highly active and ADPr is reversed by its macrodomain 1. 293T cells were transfected with the indicated plasmids in the presence or absence of PARP14 inhibitor (PARP14i). Cell lysates and GFP-immunoprecipitations (GFP-IP) were examined by western blotting using the indicated antibodies. Tubulin and Ponceau S were used as a loading controls. (D) PARP14 activity on nucleic acid substrates is not detectable in cells. U2OS cells were transfected with the indicated plasmids. gDNA or total RNA was spotted on membranes to examine the levels of ADPr. (E) Endogenous PARP14 ADP-ribosylates protein substrates in both unstimulated and IFNγ stimulated cells. A549 cells, in the presence or absence of PARP14i, were stimulated or not with IFNγ (100 ng/mL). Cell lysates were probed with the indicated antibodies. Phosopho-STAT1 was used to demonstrate IFNγ stimulation. GAPDH was used as a loading control.

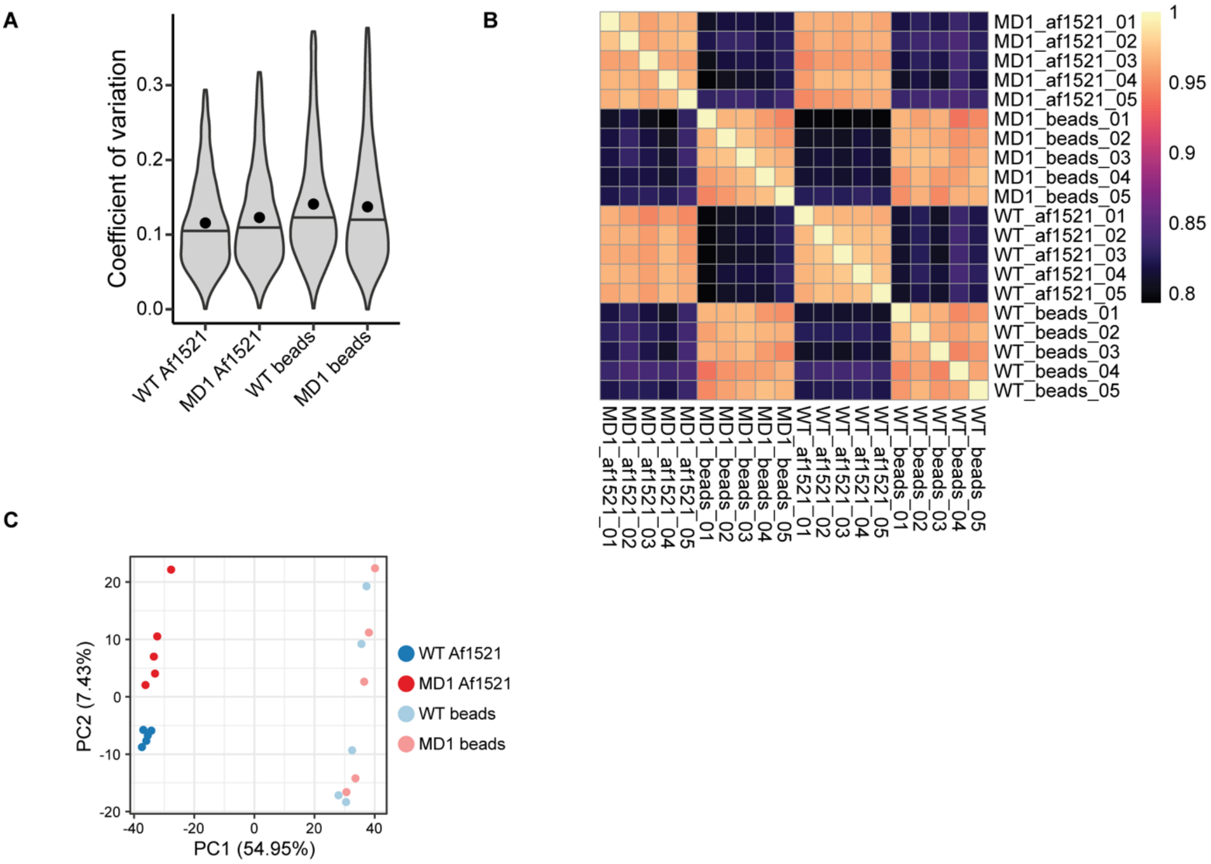

Supplementary Figure 4: Mass-Spec identification of ADP-ribosylated proteins regulated by PARP14 macrodomain 1. (A) Violin plots depicting the distribution of the coefficient of variation (CV) within the different experimental conditions. The flat line corresponds to the median CV, while the dots marks the mean CV of the distributions. Outliers were visually removed from the plot. Rows were considered to be outliers if their CV's exceed 1.5 times the inter-quantile-range (IQR) of the distribution. (B) Heatmap showing the inter-experiment Pearson correlation coefficients of the samples (C). Linear dimensionality reduction of the dataset by principal component analysis (PCA), displaying the clustering of each experiment. The x- and y-axis display the first two principal components, which capture 54.95% and 7.43% of the total variance of the dataset, respectively.

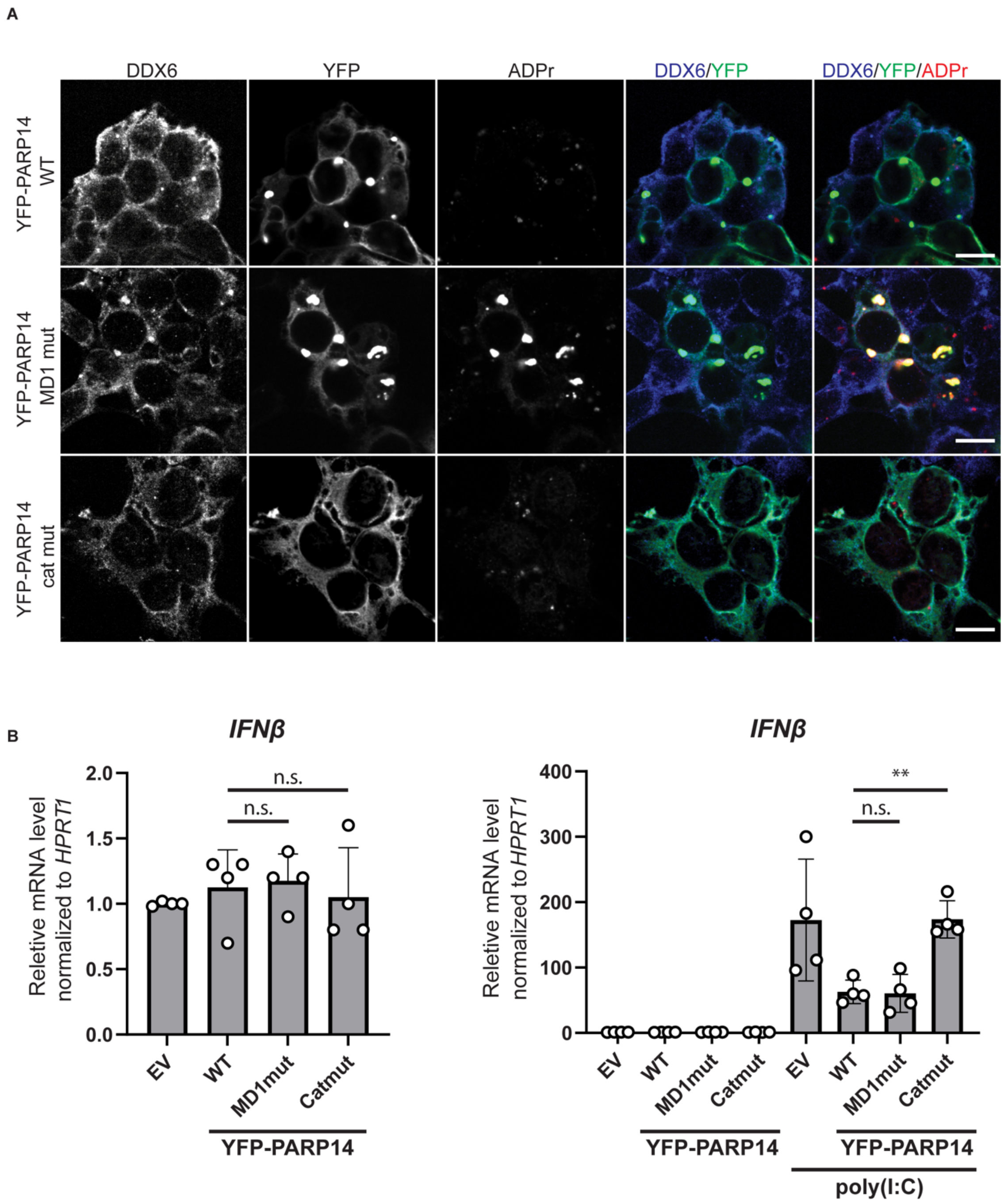

Supplementary Figure 5: PARP14 regulates various cellular functions. (A) PARP14 MD1 mutant colocalise with DDX6 and ADP-ribosylation. 293T cells were transfected with the indicated plasmids and examined by immunofluorescence with DDX6 (Blue), or ADPr (poly/mono) (red) antibodies. YFP expression is shown in Green. Scale bar: 10 μm. (B) The relative gene expression analysis of *IFNβ* in unstimulated and Poly(I:C) stimulated 293T cells transfected with PARP14 WT, PARP14 MD1 mutant or PARP14 catalytic mutant as determined by RT-qPCR normalized to the expression of *HPRT1*. Left panel: Unstimulated 293T cells. Right: 293T cells stimulated with Poly(I:C) for 24 hours. Data collected from cells without Poly(I:C) treatment is recapitulated to show the increase in expression. EV: empty vector (YFP), WT: YFP-PARP14 WT, MD1 mut: YFP- PARP14 MD1 mutant, Catmut: YFP-PARP14 catalytic domain mutant. Error bars indicate average S.D. from four independent replicates. Asterisks indicate statistical significance compared with the control, as determined by Welch's t-test (ns: not significant, ** : $p < 0.01$, Two-tailed P value, WT vs Catmut, $p = 0.0012$).

Supplementary Tables:

Supplementary Table 1. Mono-ADP-ribosylated peptides used in this study.

Peptide	Sequence
Arginine-ADPr	Ac-GR(ADPr)LIFAG-OH
Serine-ADPr	Ac-PAKS(ADPr)APAPKKG-NH_2
Glutamate-ADPr	H-AAPVE(ADPr)VVAPR-NH_2

Supplementary Table 2. Oligos used in this study.

Name	Sequence (5´-3´)
5P RNA 3Cy3	[Phos] GUGGCGCGGAGACUUAGAGAA [Cy3]
5Cy3 RNA 3P	[Cy3] GUGGCGCGGAGACUUAGAGAA [Phos]
5P DNA3 Cy3	[Phos] GTGGCGCGGAGACTTAGAGAA [Cy3]
Short21	TTCTCTAAGTCTCCGCGCCAC
cn2	TTCTCTAAGTCTCCGCGCCACTAAACCGCGCCCCTTAAGG
cn6	GGAATTCCCCGCGCCAAATTTCTCTAAGTCTCCGCGCCAC
cn7	TTTGGCGCGGGGAATTCC
cn8	ATTTGGCGCGGGGAATTCC
cn10	AAGAACAAGTCTCCGCGCCAC
cn11	TTCTCTAAGTCTCCGAGACGA
cn12	CCTTAAGGGGCGCGGTTT
cn13	CCTTAAGGGGCGCGGTTTA
ssDNA for competition assay	[Phos]GTGGCGCGGAGACTT
5P E21 ssDNA	[Phos] GTGGCGCGGAGACTTAGAGAA [Cy3]
3P E21 ssDNA	[Cy3] GTGGCGCGGAGACTTAGAGAA [Phos]
RNA oligo for RhsP2	AUCUACGGUACCUCUGGCUACGACGACAGGCGCUAAUCAGACUCCGACUG

Supplementary Table 3: Primers used in this study.

Name	Sequence (5´-3´)
PARP14 R1699A Forward	TTTAACGCCAGCTATGCCGGAAAGAATGCTGTG
PARP14 R1699A Reverse	GTCAATCGAAATGGCTTTAACGCCAGCTAT
PARP14 G832E Forward	CTTAAGCATTATGGTGAGCTGGCCGCTGCGCTCT
PARP14 G832E Reverse	AGAGCGCAGCGGCCAGCTCACCATAATGCTTAAG
PARP14 G1044E Forward	CTCGTGCTTAGTAGAGAGCCTCTTTCTAAGTCCC
PARP14 G1044E Reverse	GGGACTTAGAAAGAGGCTCTCTACTAAGCACGAGA
PARP9 G113E Forward	GCATGGGGGAGAACTGGCCCTGG
PARP9 G113E Reverse	AGAAGATCTTCATTGGCTGCATTC

Supplementary Table 4: Antibodies used in this study.

Target	Host	Company	Reference	Dilution in WB	Dilution in IF
GFP	Rabbit	Abcam	Ab290	1:3000	-
PARP14	Rabbit	Abcam	Ab229756	1:1000	-
β-tubulin	Rabbit	Abcam	Ab6046	1:2000	-
DDX3	Mouse	Proteintech	67915-1-Ig	1:1000	-
HDAC2	Mouse	Santa Cruz Biotechnology	sc-9959	1:1000	-
DDX6	Rabbit	Proteintech	14632-1-ap	1:1000	-
GAPDH	Mouse	Merck	MAB374	1:3000	-
RPA2	Rabbit	Cambridge Bioscience	A300-244A	1:2000	-
Histone H3	Rabbit	Sigma-Aldrich	06-755	1:10000	-
FLAG	Rabbit	Sigma-Aldrich	F7425	1:2000	-
autoanti-dsDNA	Mouse	DSHB	AB_10805293	1:200	-
Phosphor-STAT1 (Tyr701)	Rabbit	Cell Signaling	7649	1:1000	
DDX6	Mouse	Abnova	h00001656-m01	-	1:500
GFP	Goat	Abcam	Ab5450	-	1:500
Poly/mono	Rabbit	Cell Signaling	83732	1:1000	1:500
Anti-mouse Alexa Fluor 405	Donkey	Thermo Fisher Scientific	A48257	-	1:500
Anti-goat Alexa Fluor 488	Donkey	Thermo Fisher Scientific	A11055	-	1:500
Anti-rabbit Alexa Fluor 647	Donkey	Thermo Fisher Scientific	A32795	-	1:500
HRP conjugated anti-Mono-ADPr	-	-	Gift from Ivan Matic (*53*)	1:1000	-
HRP-conjugated anti-mouse	Goat	Agilent	P0447	1:3000	-
HRP-conjugated anti-rabbit	Swine	Agilent	P0399	1:3000	-

Supplementary Table 5: RT-qPCR primers used in this study.

Gene	Forward (5’-3’)	Reverse (5’-3’)
IFNβ	TAGCACTGGCTGGAATGAG	GTTTCGGAGGTAACCTGTAAG
HPRT1	GCGTCGTGATTAGCGATGATG	CTCGAGCAAGTCTTTCAGTCC

Other Supplementary Material for this manuscript includes:

Supplementary Data 1: Table with Af1521-enriched proteins
Supplementary Data 2: Table with MD1-specific ADPr-targets
Supplementary Data 3: Table with gene set enrichment analysis statistics
